# External exposome and all-cause mortality in European cohorts: the EXPANSE project

**DOI:** 10.3389/fepid.2024.1327218

**Published:** 2024-05-28

**Authors:** Federica Nobile, Konstantina Dimakopoulou, Christofer Åström, Fabián Coloma, Payam Dadvand, Jeroen de Bont, Kees de Hoogh, Dorina Ibi, Klea Katsouyanni, Petter Ljungman, Erik Melén, Mark Nieuwenhuijsen, Regina Pickford, Johan Nilsson Sommar, Cathryn Tonne, Roel C. H. Vermeulen, Danielle Vienneau, Jelle J. Vlaanderen, Kathrin Wolf, Evangelia Samoli, Massimo Stafoggia

**Affiliations:** ^1^ Department of Epidemiology, Lazio Region Health Service/ASL Roma 1, Rome, Italy; ^2^ Institute of Environmental Medicine, Karolinska Institutet, Stockholm, Sweden; ^3^ Department of Hygiene, Epidemiology and Medical Statistics, Medical School, National and Kapodistrian University of Athens, Athens, Greece; ^4^ Department of Public Health and Clinical Medicine, Umeå University, Umeå, Sweden; ^5^ Barcelona Institute for Global Health (ISGlobal), Barcelona, Spain; ^6^ Universitat Pompeu Fabra (UPF), Barcelona, Spain; ^7^ CIBER Epidemiología y Salud Pública (CIBERESP), Madrid, Spain; ^8^ Department of Epidemiology and Public Health, Swiss Tropical and Public Health Institute, Allschwil, Switzerland; ^9^ University of Basel, Basel, Switzerland; ^10^ Institute for Risk Assessment Sciences (IRAS), Utrecht University, Utrecht, Netherlands; ^11^ MRC Centre for Environment and Health, School of Public Health, Imperial College London, London, United Kingdom; ^12^ Department of Cardiology, Danderyd Hospital, Stockholm, Sweden; ^13^ Department of Clinical Sciences and Education, Södersjukhuset, Karolinska Institutet, Stockholm, Sweden; ^14^ Sachś Children and Youth Hospital, Södersjukhuset, Stockholm, Sweden; ^15^ Institute of Epidemiology, German Research Center for Environmental Health, Helmholtz Zentrum München, Neuherberg, Germany

**Keywords:** air pollution, air temperature, exposome, environment, green space, mortality

## Abstract

**Background:**

Many studies reported associations between long-term exposure to environmental factors and mortality; however, little is known on the combined effects of these factors and health. We aimed to evaluate the association between external exposome and all-cause mortality in large administrative and traditional adult cohorts in Europe.

**Methods:**

Data from six administrative cohorts (Catalonia, Greece, Rome, Sweden, Switzerland and the Netherlands, totaling 27,913,545 subjects) and three traditional adult cohorts (CEANS-Sweden, EPIC-NL-the Netherlands, KORA–Germany, totaling 57,653 participants) were included. Multiple exposures were assigned at the residential addresses, and were divided into three *a priori* defined domains: (1) air pollution [fine particulate matter (PM_2.5_), nitrogen dioxide (NO₂), black carbon (BC) and warm-season Ozone (warm-O_3_)]; (2) land/built environment (Normalized Difference Vegetation Index—NDVI, impervious surfaces, and distance to water); (3) air temperature (cold- and warm-season mean and standard deviation). Each domain was synthesized through Principal Component Analysis (PCA), with the aim of explaining at least 80% of its variability. Cox proportional-hazards regression models were applied and the total risk of the external exposome was estimated through the Cumulative Risk Index (CRI). The estimates were adjusted for individual- and area-level covariates.

**Results:**

More than 205 million person-years at risk and more than 3.2 million deaths were analyzed. In single-component models, IQR increases of the first principal component of the air pollution domain were associated with higher mortality [HRs ranging from 1.011 (95% CI: 1.005–1.018) for the Rome cohort to 1.076 (1.071–1.081) for the Swedish cohort]. In contrast, lower levels of the first principal component of the land/built environment domain, pointing to reduced vegetation and higher percentage of impervious surfaces, were associated with higher risks. Finally, the CRI of external exposome increased mortality for almost all cohorts. The associations found in the traditional adult cohorts were generally consistent with the results from the administrative ones, albeit without reaching statistical significance.

**Discussion:**

Various components of the external exposome, analyzed individually or in combination, were associated with increased mortality across European cohorts. This sets the stage for future research on the connections between various exposure patterns and human health, aiding in the planning of healthier cities.

## 1 Introduction

Urbanization is a rapidly growing phenomenon worldwide. In Europe, 75% of the population is currently living in urban areas in 2020 and this proportion is projected to reach 83.5% by 2050. Among its many consequences, urbanization is generally associated with poor environmental quality, including issues such as bad air quality, diminishing contact with green and blue areas, and the presence of urban heat islands.

In 2019, air pollution was estimated to be the 4th leading cause of death worldwide, surpassing other well-acknowledged risk factors for chronic non-communicable diseases such as obesity (high body-mass index), hypercholesterolemia, and malnutrition (1); furthermore, the burden of disease associated to it is large and growing (2). The adverse association between long-term exposure to air pollution, especially airborne particles, and all-cause mortality has been well-established, and numerous studies have investigated the link between various air pollutants and mortality outcomes (3–5). Long-term associations with mortality have also been found at relatively low levels of air pollution (6–8).

Moreover, accessing green and blue spaces is a challenge for residents in urban areas (9). Some studies have found beneficial associations between green spaces around the home addresses and mortality (10–13), as well as other health outcomes (14). Less is known about the relationship between blue spaces and mortality, and the few existing findings have been generally heterogeneous, partially due to differences in settings and methodologies (15, 16).

Furthermore, while the short-term effects of air temperature on mortality have been extensively investigated (17–19), studies on the long-term effects are fewer (20–22). In this context, harmful effects of diurnal temperature range (21) or increased summer mean temperature and seasonal standard deviations (20, 22) have been reported. Previous studies have also shown that the findings related to the long-term effect of temperature were modified by several factors, such as demographic characteristics and socioeconomic status (20). Additionally, a recent systematic review found clear associations between long-term exposure to unusual temperature levels and mortality, but they varied according to geographical locations (23). These environmental factors have been typically analysed separately, without considering their interplay, which collectively form part of the “external exposome” (24). This concept describes an individual's lifelong exposure to external factors and influences and encompasses, among others, the co-existing and interaction of environmental factors. Therefore, the need to focus on studies that consider subjects' exposure to several environmental variables has become evident in the last years; with this aim, various statistical methods have been applied in different contexts (25). However, greater attention has been paid to the internal exposome (26) and to specific cohorts (for instance young ages) (27).

The objective of this longitudinal study was to integrate environmental factors into *a priori* defined domains (air pollution, land/built environment and air temperature) with the aim of characterizing the external exposome and investigating its relationship with all-cause mortality. Nine cohorts across Europe were analysed, using harmonised protocols, including large administrative cohorts, that provided substantial statistical power, and smaller but better characterized traditional adult ones, allowing for adjustment of estimates based on various individual lifestyle factors.

## 2 Materials and methods

### 2.1 Study populations

Within the EXPANSE Project (EXposome Powered tools for healthy living in urbAN SEttings), which aimed to investigate a range of ambient environmental exposures and health data from multiple cohorts (28, 29), we collected data from six administrative cohorts: country-wide Greece, Sweden, Switzerland, and the Netherlands, region-wide Catalonia (Spain) and metropolitan-wide Rome (Italy); and from three traditional adult cohorts: CEANS (Cardiovascular Effects of Air Pollution and Noise in Stockholm study) in Sweden, EPIC-NL (European Prospective Investigation into Cancer and Nutrition, the Netherlands) in the Netherlands, and KORA (Cooperative Health Research in the Region of Augsburg) in Germany (pp. 2–17, Supplementary Figure S1). Briefly, the administrative cohorts are those established through record linkage e.g., with population-based archives and census registers, followed-up from one year between 2010 and 2015 until 2018 or 2019. In these cohorts we analyzed participants aged more than 37 years old, to match the availability of data in the Greek one. The three traditional adult cohorts were enrolled in one year between 1992 and 2001 and followed-up until one year between 2011 and 2016, without any specific age restrictions. In both sets of cohorts, subjects were followed-up until death from all-causes (ICD-10: A00-Z99), emigration out of the study area, or end of the study, whichever came first. Participants without a geocoded residential address at baseline were excluded. All-cause mortality was analyzed as health outcome, due to its minimal potential for misclassification, rather than cause-specific mortality (30).

For each cohort, baseline information on age, sex, and individual socio-demographic characteristics were available. Furthermore, we recorded area-level variables, such as socio-economic status indicators (e.g., deprivation index, unemployment rate, mean income, etc.), based on their availability in each cohort. The data records were extracted in accordance with a shared codebook. The analyses were conducted locally by each cohort manager using a common script, and the results were centrally shared with the Department of Epidemiology (ASL Roma 1, Rome, Italy).

This study was carried out following the guidelines of the Declaration of Helsinki. Each cohort received approval from the respective institutional review boards or local/regional legislation, ensuring compliance with all applicable national, state, and local regulations. Informed consent was obtained from each participant of the three traditional cohorts.

### 2.2 Exposure domains

Each exposure variable, in terms of annual average value, was assigned to participants' residential addresses at baseline within each cohort, following a harmonized European-wide exposure assessment protocol established for all studies developed in the EXPANSE Project (Supplementary Table S1). Subsequently, these exposures were *a priori* grouped into three domains: air pollution, land/built environment and air temperature.

#### 2.2.1 Air pollution domain

In the air pollution domain, four exposures were included: particulate matter with an aerodynamic diameter <2.5 µm (PM_2.5_), nitrogen dioxide (NO_2_), black carbon (BC), and ozone related to warm season—from April to September (O_3warm_). They were estimated through western Europe-wide land-use regression models for the year 2010 on a grid of 100 m × 100 m. The modelling process is described elsewhere (31). Briefly, concentrations measured at site-level by the AirBase network of the European Environmental Agency (EEA) or during purpose designed monitoring campaigns were used as target variables, and satellite data, dispersion model estimates, road network data and land-use characteristics were used as spatial predictors. The performance of the models was assessed by a 5-fold hold out validation: the R^2^ (percentage of the explained variable) in left-out observations was equal to 66% for PM_2.5_, 58% for NO_2_, 51% for BC and 60% for O_3warm_.

#### 2.2.2 Land/built environment domain

In the land/built environment domain three exposures were included: green spaces, impervious surfaces (as a measure of built-up areas or gray spaces) and blue spaces. The green spaces indicator was determined by the satellite-based Normalized Difference Vegetation Index (NDVI) measure to assess the presence of vegetation. The Vegetation Indices (MOD13Q1) product of the Terra Moderate Resolution Imaging Spectroradiometer (MODIS) with a resolution of 250 m × 250 m in 2019 (32) was used.

Impervious surfaces were defined as areas that were sealed or covered by materials that prevent natural water infiltration into the soil, such as roads, buildings, parking lots, and other artificial structures. The indicator was obtained from the Copernicus Land Monitoring Service (CLMS) for 2015 available as a percentage in cells of 100 m × 100 m (33).

Exposure to blues spaces was defined as the distance to the closest body of water (i.e., rivers, lakes, sea). This indicator corresponded to Euclidian distance in meters between the centroid of the cells of 100 m × 100 m where the residential address was located and the blue space, assessing it through the EU-Hydro map from the CLMS for 2011 (34).

#### 2.2.3 Air temperature domain

In the air temperature domain, four variables were included: mean and standard deviation (SD) of air temperature in the warm season (April-September) and mean and SD of air temperature in the cold season (October-March). They were obtained from the daily air temperature data estimated within the ERA5-Land reanalysis dataset for 2010. This repository was available with a resolution of 0.1° × 0.1° (Celsius degrees), which corresponds to ∼11km × ∼11 km in Europe.

### 2.3 Statistical analysis

The statistical analysis was conducted in several stages and applied within each cohort.

In the first step, Principal Component Analysis (PCA) was performed separately in each *a priori* defined exposure domain. This methodology has been widely used in recent literature (35–37) and allows to synthesize the multiple variables of the domain into a reduced set of independent principal components (PCs), e.g., linear combinations of the original variables. Such PCs were arranged in descending order of variance and were mutually orthogonal. With the aim of preserving as much of the reported information as possible, we retained as many PCs as to capture at least 80% of the explained variability. This resulted in one or two PCs per cohort for each domain. Furthermore, the contribution of each exposure variable on each PC and their correlation were investigated, in order to facilitate interpretation of the PCs. In fact, we conducted PCA within the *a priori* defined domains, rather than considering all exposure variables together, in order to obtain more interpretable and comparable PCs across cohorts.

In the second step, Cox proportional hazard models were applied, using the selected PC(s) as continuous exposure variables and all-cause mortality as outcome. Three progressively more detailed adjustment structures were established, based on approaches used in previous studies (6, 29, 38). The specific covariates included in the models varied according to administrative and traditional adult cohorts data availability: (1) age (as time scale), sex (as strata term), a sub-cohort indicator (as strata term, if applicable), year of enrollment (for adult cohorts), and a dummy variable for large region (NUTS-1, for country-wide cohorts); (2) covariates from model 1 and all available individual-level variables at baseline (e.g., marital status, educational level, smoking status, etc.); (3) covariates from model 2 and several area-level variables used to characterize the residential neighborhood, e.g., socio-economic status. Within cohort, all analyses, regardless of the adjustment model, were performed on a dataset with non-missing values for all covariates related to model 3 (considered the main model) to allow comparisons across models.

Initially, single-component-exposure models were applied. Subsequently, two-component-exposure models within each domain and multi-component models with all selected PCs were considered for each cohort.

Finally, the Cumulative Risk Index (CRI), that sums the variable-specific effect estimates to an overall risk through multi-component-exposure models, was calculated to summarize the risk related to each domain (from the selected PCs within domain) and to the overall external exposome (from all the selected PCs across domains) (39). In other words, the CRI represents the additive effects (on the log-scale) of the joint exposure variables, obtained by summarizing the single effect of each exposure, adjusted for the other exposures included in the survival model.

The estimates were expressed as Hazard Ratios (HRs) with 95% confidence interval (95% CI) per one interquartile (IQR) changes: within each cohort, we used increases in the IQRs, while we used decreases for the first PC of land/built environment domain related to higher vegetation/lower anthropogenic surfaces, to identify it as risk factor for health.

Sensitivity analyses were conducted by excluding the temperature domain for two main reasons: the cruder spatial resolution of the included variables compared to those of the two other domains, and the highly variable results across cohorts, limiting interpretability.

The data were analyzed by each cohort through a standardized R script (R version 4.0.0), including the following packages: *FactoMineR*, *survival*, *ggplot2*.

## 3 Results

A total of 29,462,905 participants were enrolled in the six administrative cohorts, and 63,234 in the three traditional adult cohorts. The complete datasets for model 3 consisted of 27,913,545 and 57,653 subjects, respectively. These participants contributed 204,082,807 person-years and 1,019,175 person-years (p-y) during the follow-up periods, resulting in 3,253,047 and 5,605 deaths (Table 1). Among the administrative cohorts, Greece had the highest mortality rate per 1,000 p-y at 21.7, and among the traditional adult cohorts KORA with a rate of 11.8. The mean age varied from 49.8 (SD = 13.9) in the KORA cohort to 60.3 (SD = 15.3) in the Greek cohort, while the percentage of females ranged from 48.4% in Sweden to 77.1% in the EPIC-NL cohort.

**Table 1:** Description of the European cohorts (six administrative and three traditional adult cohorts) and participants characteristics (complete case on individual- and area-level covariates of model 3).

	Administrative cohort						Traditional adult cohort		
	Catalonia cohort	Greece cohort	Rome cohort	Sweden cohort	Switzerland cohort	Netherlands cohort	CEANS cohort	EPIC-NL cohort	KORA cohort
Participants	3,640,594	6,121,421	1,539,784	4,274,326	4,427,855	7,909,565	19,888	29,428	8,337
Recruitment	2015	2014	2011	2010	2011	2010	1992	1993	1994–2001
End of follow-up	2019	2019	2019	2018	2019	2019	2011	2016	2011–2014
Female (%)	52.8	52.5	55.3	48.4	52.0	50.6	57.8	77.1	50.5
Age (year) at baseline [mean (SD)]	56.7 (14.1)	60.3 (15.3)	59.3 (14.5)	59.3 (13.9)	57.7 (13.5)	53.3 (13.2)	56.4 (11.4)	50.6 (11.2)	49.8 (13.9)
Person-years at risk	17,475,621	29,141,748	11,463,921	35,835,230	36,457,152	73,709,135	259,019	619,572	140,583
Deaths	182,788	632,439	201,093	618,983	553,107	1,064,637	3,036	912	1,657
All-cause mortality rate per 1,000 p-y	10.5	21.7	17.5	17.3	15.2	14.4	11.7	1.5	11.8

### 3.1 Principal component analysis

Based on the criterion of capturing 80% of the variability, two PCs from the air pollution domain were selected for Greece (89% of the explained variability), Sweden (93%), Switzerland (89%), the Netherlands (90%), CEANS (89%), and EPIC-NL (90%) and one for Catalonia (92%), Rome (83%) and KORA cohorts (82%) (Table 2). Across all the cohorts, the four air pollutants generally exhibited similar contributions to the first PC, except for O_3_ in Greece and Sweden and PM_2.5_ in EPIC-NL cohort, that had an almost negligible contribution. Positive loadings were observed for PM_2.5_, NO_2_, and BC, while negative loadings for O_3_. Additionally, the main contribution to the second PC came from O_3_, with a positive loading, for Greece, Sweden, and Switzerland, and from PM_2.5_, with a positive loading, for the Netherlands, CEANS and EPIC-NL cohort (Supplementary Figures S2, S3). The estimates were expressed as HRs per IQR increases for each PC.

**Table 2:** Explained variance (%) and descriptive statistics [median (IQR)] of selected principal components (PCs) for each cohort and domain.

	Air pollution		Land/built environment		Air temperature	
	1^a^ PC	2^a^ PC	1^a^ PC	2^a^ PC	1^a^ PC	2^a^ PC
Catalonia	92.1%; 0.041 (3.137)	–	58.2%; −0.344 (1.604)	31.5%; −0.213 (1.243)	69.6%; −0.522 (2.027)	28.4%; −0.200 (0.429)
Greece	61.6%; −0.267 (2.595)	27.2%; 0.236 (1.574)	60.1%; −0.426 (1.841)	33.3%; −0.287 (1.673)	67.8%; 0.623 (2.222)	24.6%; 0.425 (1.453)
Rome	83.2%; −0.002 (2.187)	–	57.5%; −0.155 (1.893)	32.5%; −0.279 (1.405)	75.9%; −0.019 (2.631)	23.5%; 0.505 (0.946)
Sweden	64.9%; −0.031 (2.139)	28.2%; −0.040 (1.375)	58.8%; 0.238 (1.725)	32.4%; −0.228 (1.113)	67.4%; 0.332 (1.505)	23.0%; 0.082 (1.530)
Switzerland	78.6%; −0.057 (2.042)	10.3%; −0.083 (0.656)	61.3%; 0.000 (1.706)	28.5%; −0.157 (1.082)	74.0%; 0.353 (0.925)	20.6%; 0.185 (1.011)
Netherlands	74.0%; −0.023 (2.128)	16.1%; 0.018 (1.113)	58.0%; −0.107 (1.705)	29.2%; −0.134 (1.084)	77.7%; 0.280 (3.082)	21.4%; 0.057 (0.940)
CEANS	74.5%; −0.041 (2.328)	14.5%; 0.080 (0.875)	59.7%; 0.283 (1.713)	31.7%; −0.194 (1.186)	90.9%; 0.017 (2.814)	–
EPIC-NL	62.6%; −0.274 (2.112)	27.2%; −0.167 (1.347)	57.3%; −0.092 (1.628)	29.7%; −0.144 (1.067)	93.6%; −0.410 (2.885)	–
KORA	81.9%; −0.302 (2.190)	–	64.4%; −0.173 (1.784)	24.1%; −0.091 (0.924)	68.9%; −0.874 (1.960)	27.6%; −0.508 (1.481)

In the land/built environment domain, two PCs were selected for all cohorts, explaining variances from 87% to 93% (Table 2). This domain exhibited the most consistent pattern across all the cohorts. Specifically, green spaces and impervious surfaces were major contributors to the first PC, while blue spaces played a significant role in the second one. Higher levels of NDVI and lower levels of impervious surfaces correlated with higher first component values (Supplementary Figures S4, S5). With the aim to identify it as a risk factor, the estimates of the HR were expressed per IQR decreases of the component for all the cohorts. Moreover, a greater distance from the water was associated with higher values of the second PC.

Finally, two PCs were extracted for all cohorts in the air temperature domain, explaining variances between 90% and 99%, except for CEANS and EPIC-NL, where one PC was considered with explained variances of 91% and 95%, respectively (Table 2). All four temperature variables, albeit with varying magnitudes, contributed to the first PC. The observed loadings differed among the cohorts: they were positive for variables representing seasonal means and negative for those representing SD values for Catalonia, Rome, and Sweden; they were negative for seasonal means but positive for SD values for Greece and Switzerland; all loadings were positive for the KORA cohort, and for the Netherlands, CEANS, and EPIC-NL cohorts, except for the mean of the cold months. In contrast, the primary contribution to the second component, when selected, was derived from the mean of the warm season for Catalonia, Greece, Rome, and the Netherlands, the SD of the warm season for Sweden and Switzerland, and the SD of the cold season for the KORA cohort. In all cases, the respective observed loadings were positive (Supplementary Figures S5, S7). The estimates were expressed as HRs per IQR increases for each selected PC.

Within each cohort, the Pearson correlation coefficients between PCs in different domains were always within ±0.7 (Supplementary Figure S8).

### 3.2 Single-component-exposure models

Focusing on the main models (model 3), higher levels of the first PC in the air pollution domain were associated with increased all-cause mortality for the administrative cohorts, with Hazard Ratios (HRs) ranging from 1.011 (95% CI: 1.005–1.018) for the Rome cohort to 1.076 (95% CI: 1.071–1.081) for the Swedish cohort per cohort-specific IQR increases (Table 3). The second component, selected for Greece, Sweden, and the Netherlands, showed significant negative associations with mortality. Notably, the pollutant predominantly contributing to the latter component in all four cohorts was O_3warm_, together with PM_2.5_ only for the Netherlands.

**Table 3:** Association between principal components (PCs) explaining more than 80% variability and all-cause mortality across the European cohorts (six administrative and three traditional adult cohorts) from single-component-exposure Cox proportional hazard models. Hazard Ratios (HRs) with 95% confidence interval (95% CI) per IQR increases adjusted for available individual and area-level covariates (more details in the footnote).

	Air pollution		Land/built environment		Air temperature	
	1^a^ PC	2^a^ PC	1^a^ PC	2^a^ PC	1^a^ PC	2^a^ PC
Catalonia	1.066	–	0.994*	0.980	0.955	1.015
	(1.047–1.086)		(0.987–1.001)	(0.973–0.987)	(0.941–0.968)	(1.007–1.023)
Greece	1.044	0.976	1.022*	0.985	1.007	1.037
	(1.037–1.052)	(0.971–0.981)	(1.018–1.026)	(0.981–0.99)	(1.001–1.012)	(1.033–1.042)
Rome	1.011	–	1.016*	1.012^§^	1.004	1.008
	(1.005–1.018)		(1.009–1.023)	(1.004–1.019)	(0.997–1.012)	(1.003–1.013)
Sweden	1.076	0.957	1.069*	1.008	0.997	1.001
	(1.071–1.081)	(0.953–0.961)	(1.066–1.073)	(1.005–1.011)	(0.992–1.001)	(0.996–1.006)
Switzerland	1.050	0.998	1.078*	1.033	0.999	0.994
	(1.045–1.055)	(0.994–1.002)	(1.074–1.082)	(1.030–1.037)	(0.997–1.001)	(0.990–0.999)
Netherlands	1.027	0.982	1.006*	1.002	1.003	1.007
	(1.024–1.030)	(0.979–0.985)	(1.003–1.009)	(0.999–1.004)	(0.998–1.008)	(1.003–1.010)
CEANS	1.036	0.985	1.043*	1.039	0.984	–
	(0.978–1.097)	(0.943–1.029)	(0.986–1.103)	(0.987–1.094)	(0.916–1.057)	
EPIC-NL	0.994	1.020	0.953*	1.005	1.065	–
	(0.906–1.091)	(0.916–1.137)	(0.873–1.040)	(0.927–1.089)	(0.934–1.215)	
KORA	0.931	–	1.014*	0.982	0.993	0.998
	(0.858–1.011)	–	(0.937–1.098)	(0.932–1.036)	(0.924–1.067)	(0.923–1.079)

Catalonia: HRs adjusted for age (timescale), sex (strata), smoking status, individual income, psca index, percentage of non-Spanish residents in census tract, and population density per m^2^.

Greece: HRs adjusted for age (time scale), sex (strata), NUTS1 areas country-wide (4 levels: Attica / Aegean Islands, Crete / North Greece / Central Greece) & 4 area-level variables: tertiary education rate, unemployment rate, degree of urbanicity in 3 categories: 1. Cities (densely populated areas), 2. Towns and Suburbs (intermediate density areas) and 3. Rural areas (thinly populated areas) and married rate. For the Greater Area of Athens and other large municipalities (population greater than 100,000 inhabitants) in Greece, the aforementioned variables were available at square-block level. For the rest of the areas in Greece, the variables were available at municipality unit level.

Rome: HRs adjusted for age (time scale), sex (strata), place of birth, education level, employment status, marital status, citizenship, deprivation index on a census block level and unemployment rate, percentage of graduates and house prices on a neighborhood level.

Sweden: HRs adjusted for age (time scale), sex (strata), living condition, education level, district mean income, portion of people with high school or higher education in district, area.

Switzerland: HRs adjusted for age (time scale), strata(sex), area (i.e., 7 Swiss regions), marital status, occupational status, origin (i.e., Swiss vs. other), language region, socio-economic position index (SEP), community-level SEP index and community-level unemployment rate.

The Netherlands: HRs adjusted for age (time scale), sex (strata), area, wealth at 2010, categorized in deciles, partner status at 2010, individual socioeconomical status, area-level socio-economic status, area-level mean income at 2010, percentage of low-income households, urbanicity.

CEANS: HRs adjusted for subcohort (strata), age (timescale), sex (strata), and year of baseline visit, marital status, body-mass index, smoking (status, duration, intensity, intensity squared), employment status, education, and area-level socioeconomic status (2001 mean income on a neighborhood level).

EPIC-NL: HRs adjusted for subcohort (strata), age (timescale), sex (strata), and year of baseline visit, marital status, body-mass index, smoking (status, duration, intensity, intensity squared), employment status, education, and area-level socioeconomic status (2001 mean income on a neighborhood level).

KORA: HRs adjusted for subcohort (strata), age (timescale), sex (strata), and year of baseline visit, marital status, body-mass index, smoking (status, duration, intensity, intensity squared), employment status, education, and area-level socioeconomic status (Percentage of households with low income per 5 km^2^ grid cell in 2007).

* HRs are expressed per IQR decreases.

^§^ HR is expressed per IQR decreases because the PC is dominated by the variable related to the blue area space, and in Rome, it is driven by the Tiber River, which flows from North to South, cutting the city in the center. Therefore, higher values of the PC mean a greater distance from the center.

Lower levels of the first PC (indicating lower values of NDVI and higher values of impervious surfaces) in the land/built environment domain were associated with increased risks, showing HRs equaled to 1.022 (95% CI: 1.018–1.026), 1.016 (95% CI: 1.009–1.023), 1.069 (95% CI: 1.066–1.073), 1.078 (95% CI: 1.074–1.082), and 1.006 (95% CI: 1.003–1.009) per IQR decreases for the Greece, Rome, Sweden, Switzerland, and the Netherlands cohorts respectively, while for the Catalonia cohort the association was null [HR = 0.994 (95% CI: 0.987–1.001)]. The results for the second component (larger distance to blue spaces) were more heterogeneous among cohorts, with significant negative estimates for the Catalan and Greek cohorts and significant positive estimates for the Swedish and Swiss ones per IQR increases. For the Roman cohort, lower levels of the component (smaller distance to blue spaces) were associated with higher all-cause mortality.

The associations between the two PCs from the air temperature domain varied among cohorts and, in some cases, were not statistically significant. One-IQR increase of the first PC of this domain was associated with lower HRs of all-cause mortality for Catalonia [HR = 0.955 (95% CI: 0.941–0.968)] and higher HRs for Greece [HR = 1.007 (95% CI: 1.001–1.012)]. In contrast, higher levels of the second PC (indicating higher temperatures during warm season) were associated with higher risks for Catalonia, Greece, Rome, and the Netherlands [HR = 1.015 (95% CI: 1.007–1.023), HR = 1.037 (95% CI: 1.033–1.042), HR = 1.008 (95% CI: 1.003–1.013), HR = 1.007 (95% CI: 1.003–1.010), respectively].

The associations observed for the traditional adult cohorts (Table 3) generally aligned with the findings for the administrative cohorts, although they did not reach statistical significance. In particular, when comparing the CEANS cohort with the Swedish administrative cohort, consistent associations between the selected PCs and mortality were observed. However, the risk estimates for CEANS were generally more modest and did not attain statistical significance. A positive association was estimated for the KORA cohort between lower levels of the first component in the land/built environment domain and increased mortality [HR = 1.014 (95% CI: 0.937–1.098)] and for the EPIC-NL cohort for the first component in the air temperature domain [HR = 1.065 (95% CI: 0.934–1.215)].

The associations estimated from models 1, 2 and 3 are showed in Supplementary Figure S9. The results were quite consistent; however, regarding the first principal component of the air pollution domain, they generally became stronger, especially when area-level covariates were added in model 3.

**Figure 1:**
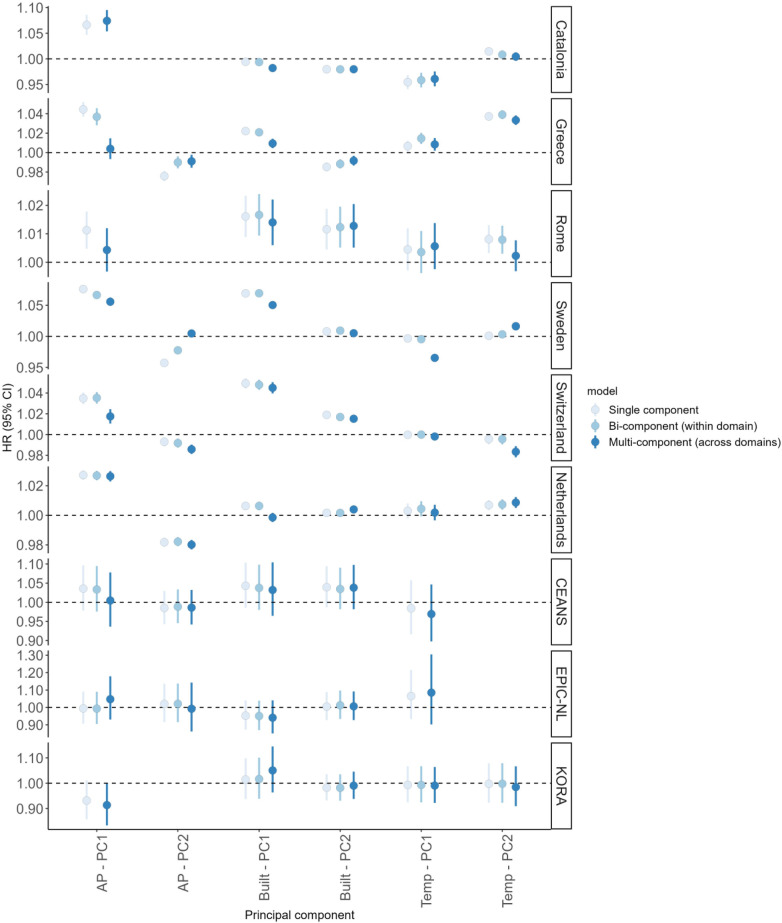
Association between principal components (PCs) and all-cause mortality across the European cohorts (six administrative and three traditional adult cohorts) from single-component-exposure, two-component-exposure (within domain, where applicable) and multi-component-exposure (across domains) Cox proportional hazard models. Hazard Ratios (HRs) with 95% confidence interval (95% CI) per IQR increases adjusted for available individual and area-level covariates (model 3). HRs of *Built—PC1* for all cohorts and *Built—PC2* for Rome cohort are expressed per IQR decreases.

The estimated associations between PCs and all-cause mortality remained unchanged after adjusting for the other component within the same domain (in cohorts where two PCs were selected for a domain), because of the orthogonality (i.e., independence) between the PCs of the same domain (Figure 1). When adjusting for all other components in multi-component-exposure models, the associations displayed distinct patterns among the administrative cohorts, with a general decrease in the HRs observed for the first PC in both the air pollution and land/built environment domains. Conversely, the associations remained relatively consistent and not statistically significant across the traditional adult cohorts.

### 3.3 Cumulative risks

Cumulative risks were estimated for each domain (if more than one PC was selected), through the CRI based on two-component-exposure models (Table 4).

**Table 4:** Cumulative risk Index (CRI) estimates for each domain and for all domains simultaneously across the European cohorts (six administrative and three traditional adult cohorts). Hazard Ratios (HRs) with 95% confidence interval (95% CI) adjusted for available individual and area-level covariates (model 3).

	Air pollution	Land/built environment	Air temperature	Air pollution + land/built environment (sensitivity)	Air pollution + land/built environment + Air temperature
Catalonia	1.066^a^	0.973	0.966	1.042	0.997
	(1.047–1.086)	(0.964–0.983)	(0.948–0.985)	(1.022–1.062)	(0.971–1.025)
Greece	1.026	1.009	1.054	1.021	1.038
	(1.013–1.040)	(1.002–1.016)	(1.046–1.062)	(1.007–1.034)	(1.023–1.052)
Rome	1.011^a^	1.029	1.011	1.033	1.040
	(1.005–1.018)	(1.019–1.04)	(1.003–1.020)	(1.022–1.045)	(1.025–1.054)
Sweden	1.043	1.079	0.999	1.085	1.099
	(1.035–1.050)	(1.075–1.084)	(0.993–1.004)	(1.075–1.094)	(1.088–1.109)
Switzerland	1.047	1.108	0.993	1.106	1.085
	(1.040–1.053)	(1.102–1.113)	(0.989–0.998)	(1.098–1.114)	(1.076–1.094)
Netherlands	1.009	1.008	1.011	1.012	1.019
	(1.004–1.013)	(1.004–1.012)	(1.005–1.018)	(1.006–1.017)	(1.011–1.027)
CEANS	1.022	1.073	0.982^a^	1.063	1.029
	(0.945–1.104)	(0.999–1.153)	(0.902–1.068)	(0.968–1.167)	(0.910–1.163)
EPIC-NL	1.014	0.963	1.065^a^	0.998	1.069
	(0.883–1.164)	(0.863–1.074)	(0.934–1.215)	(0.856–1.162)	(0.861–1.326)
KORA	0.931^a^	0.998	0.991	0.951	0.927
	(0.857–1.011)	(0.91–1.094)	(0.892–1.101)	(0.856–1.056)	(0.797–1.079)

^a^ Estimates from single-exposure models.

Among the administrative cohorts, statistically significant positive associations were found between the air pollution domain and all-cause mortality. The CRI estimates for Greece, Sweden, Switzerland, and the Netherlands were 1.026 (95% CI: 1.013–1.040), 1.043 (95% CI: 1.035–1.050), 1.047 (95% CI: 1.040–1.053), and 1.009 (95% CI: 1.004–1.013), respectively.

Positive significant associations were observed for the land/built environment domain, except for Catalonia [HR = 0.973 (95% CI: 0.964–0.983)]. The highest CRI was estimated in Switzerland [HR = 1.108 (95% CI: 1.102–1.113)], while the lowest in the Netherlands [HR = 1.008 (95% CI: 1.004–1.012)].

Concerning the air temperature domain, the CRI estimates showed a statistically significant negative association for Catalonia [HR = 0.966 (95% CI: 0.948–0.985)] and Switzerland [HR = 0.993 (95% CI: 0.989–0.998)], and positive significant associations for Greece [HR = 1.054 (95% CI: 1.046–1.062)], Rome [HR = 1.011 (95% CI: 1.003–1.020)] and, the Netherlands [HR = 1.011 (95% CI: 1.005–1.018)], while the estimates were null for Sweden.

The CRI of the external exposome for each cohort, based on the selected PCs from the three domains, was estimated as a combination of PC results from multi-component-exposure models. Positive significant associations were found between the external exposome and all-cause mortality, with HRs ranging from 1.019 (95% CI: 1.011–1.027) for the Netherlands cohort to 1.099 (95% CI: 1.088–1.109) for the Swedish cohort, per increases in the risk factors (obtained as increases in all the PCs, except for decreases in the first component of the land/built environment domain, as previously explained).

Among the traditional adult cohorts, indicative positive associations were found between all-cause mortality and both the air pollution and land/built environment domains for CEANS, and the air pollution and air temperature domains for EPIC-NL. The CRI estimates of the external exposome were 1.029 (95% CI: 0.910–1.163) for CEANS and 1.069 (95% CI: 0.861–1.326) for EPIC-NL.

Finally, in the sensitivity analysis, the air temperature domain was excluded (Supplementary Figure S10). The CRI estimates, only based on air pollution and land/built environment domains, were positive and higher than the total CRI when considering all three domains for Catalonia [HR = 1.042 (95% CI: 1.022–1.062)] and Switzerland [HR = 1.106 (95% CI: 1.098–1.114)] and lower for Greece [HR = 1.021 (95% CI: 1.007–1.034)], Rome [HR = 1.033 (95% CI: 1.022–1.045)], Sweden [HR = 1.085 (95% CI: 1.075–1.094)], and the Netherlands [HR = 1.012 (95% CI: 1.006–1.017)] (Table 4, 5th column). It was higher also for CEANS, even if not statistically significant [HR = 1.063 (95% CI: 0.968–1.167)], and for KORA, but negative and not statistically significant. Removing the effect from the air temperature domain, the CRI became negative and not statistically significant for EPIC-NL.

## 4 Discussion

In nine European cohorts, we investigated the association between the external exposome, using a combination of variables related to air pollution, land/built environment, and air temperature, and all-cause mortality. In six administrative cohorts increases in mortality with lower air quality and land/built environment were observed, both in the single-component-exposure model and CRI estimates. In three traditional adult cohorts, there were some indications of adverse associations, even if the estimates were generally not statistically significant.

To the best of our knowledge, this is the first study to investigate the association between the external exposome and all-cause mortality in multiple administrative and traditional adult cohorts. The research in the field of mapping the exposome is emerging (40), however, most studies have focused on birth or child cohorts and on its associations with different outcomes (41, 42).

### 4.1 Single-component-exposure results

Recent systematic reviews and meta-analyses found that ambient PM_2.5_, PM_10_, NO_2_ and O_3_ are significantly associated with all-cause mortality (30, 43, 44). In our analyses we used PCA to synthesize multiple air pollutants into a reduced set of PCs, rather than looking at single pollutants, since people are exposed to a mix of ambient air pollutants. Even if not directly comparable, for the administrative cohorts our results are in line with the results of the meta-analyses, showing overall small positive associations between exposure to the first PC of air pollution domain and all-cause mortality.

In all cohorts, the land/built environment domain was characterized by decreases in green spaces, increases in built up areas, and greater distance from water bodies. An accumulating body of evidence suggests beneficial health effects of larger green areas, in contrast to excessive built-up areas (12, 13, 45), through mechanisms such as stress reduction, mitigation of extreme temperatures, pollution absorption, and opportunities for physical activity and social interactions. In line with the available evidence, we found that reduced exposure to vegetated areas and increased built-up land covers are associated with increased mortality. Although studies on distance to blue spaces and health are still limited, some mechanisms for a positive impact of blue spaces on health have been proposed. In a systematic review and meta-analysis, Georgiou et al. (46) suggest four mediating pathways: physical activity, restoration, social interaction and better environmental co-factors. In our analysis, only the Catalonia cohort showed a negative association between the overall land/built environment domain and mortality, specifically due to a greater distance from water being associated with a decreased mortality risk. This finding is consistent with the results of Nieuwenhuijsen et al. (2018) (16), who observed statistically significant increased risks of mortality with a higher percentage of blue spaces in a cohort from Barcelona (Spain). They hypothesized that blue spaces could act as a proxy for other exposures, such as emissions from ships, ports and other chemicals.

There are yet to be clear hypotheses established regarding how long-term exposure to non-optimal ambient temperature might impact all-cause mortality. Compared to long-term average air pollutants and the land/built environment, ambient temperature is more variable and hence a long-term impact on human health might be difficult to capture. This is even more true in nationwide cohorts, where average temperature levels might correlate with urban structures, and act as a proxy for urban-rural differences. With few exceptions, our data showed mostly no association for the air temperature domain. Given the complexity of the interpretation, we consider these results far from being conclusive, and therefore we added a sensitivity analysis where the air temperature domain was entirely removed. Overall, the external exposome (e.g., the combination of all the three environmental domains under investigation) showed a positive association with mortality in all administrative cohorts, except for Catalonia. However, when excluding the air temperature domain, the association between the external exposome and mortality became positive and statistically significant also in Catalonia. Regarding the traditional adult cohorts, signals of positive associations were found in all cohorts, except for KORA, but results in the traditional cohorts were highly unstable due to reduced statistical power.

### 4.2 Cumulative risks results

The use of the PCA allowed for an initial dimensionality reduction, synthesizing multiple environmental risk factors into a limited number of components that partitioned the original variance into few independent and interpretable components.

In a following step, based on the two-component-exposure models within the same domain, when applicable, we computed the cumulative risks of air pollution, land/built environment and air temperature domain. The obtained CRI estimates, integrating different sources, could provide a more comprehensive assessment of the environmental stressors in a compact and more comprehensible way.

Our results indicated an increase in mortality associated with elevated air pollution levels. However, for instance, in the case of two selected components, the total risk estimates for the domain were generally more modest compared to those of the first PC alone, which mainly arose from the combination of effects of PM_2.5_, NO_2_, and BC. This is due to the HRs of the second PC, which were lower than 1 and predominantly driven by the often protective effects of O_3_. The negative estimates related to O_3_ are consistent with several existing studies on European cohorts (44).

Regarding the land/built environment domain, lower green spaces and higher built-up areas (first PC), along with greater distance from water (except for Rome, where shorter distance was considered a risk factor), were associated with higher mortality. The HRs of the domain across cohorts were stronger than those obtained by considering only NDVI and impervious surfaces or distance from water. As previously discussed, Catalonia, EPIC-NL, and KORA deviated from these general findings, due to different effects for the presence of water bodies and not statistically significant results.

The non-uniform and generally statistically non-significant results of the PCs within the air temperature domain resulted in null HR of the CRI, except for the positive estimates in Greece, Rome, and the Netherlands.

The health of an individual is greatly influenced by the complex interplay between genetic factors and external environmental stressors (47) and, for instance, the effect of one exposure can mitigate or magnify the effect of another. Unraveling the biological mechanisms that underly these associations and its impact on health outcomes has highlighted the importance of considering the exposome approach, which moves beyond analyzing individual exposures (48). The advantage of using a composite score is the reduction of multiple correlated estimates into a single one. This way, the results of the analysis are easier to interpret and communicate. These analyses showed an increased risk for all-cause mortality for the combination of higher air pollution and poorer land/built environment for almost all the cohorts included in the analysis. Associations for the CRI were generally higher than those for the single exposures, suggesting that interaction between different environmental stressors might play a role. Thus, these results imply the need to implement policies aimed at improving various aspects of the external exposome, such as reducing air pollution and anthropogenic areas while increasing urban green spaces.

### 4.3 Strengths and limitations

This study has some limitations. First, lifestyle individual-level covariates, such as smoking or body mass index, were not available for the administrative cohorts, preventing accurate control for individual-level confounding there. However, we compensated the lack of lifestyle covariates with data on contextual characteristics, specifically on socio-economic status. Also, previous studies on air pollution, using causal diagrams, showed that, in case of external exposures, strong residual confounding from omitted individual-level covariates is not expected, once area-level ones are duly accounted for (49). In addition, we included in the analyses three adult cohorts with extensive individual-level covariates.

Second, the air temperature domain consisted of variables with coarser spatial resolution, compared to the other included exposome factors, which may have introduced exposure measurement error and reduced spatial contrast, especially in the traditional adult cohorts which mostly resided within a single city or metropolitan area. Furthermore, the epidemiological evidence and the biological plausibility of adverse effect of long-term exposure to air temperature on human mortality are still uncertain. For these reasons, we conducted a sensitivity analysis excluding the air temperature domain and found consistent adverse associations of the remaining domains with mortality.

A third limitation of our study was the relatively modest number of exposure variables in each domain and the small number of domains defining the external exposome. The first aspect might have produced weak PCs, while the second might have neglected other potentially important aspects of the external exposome, such as contextual covariates related to diet, lifestyle, and other environmental contaminants. However, this study stems from the need to provide initial results, in a pilot framework, on exposome-approach analyses with harmonized data and methodologies. We believe that our results, although partial, still provide new insights on the interplay between the selected domains and mortality risks. Future analyses should expand our approach by incorporating additional exposures within the EXPANSE project.

Furthermore, all the exposures used in our analysis were assigned at residential addresses at baseline, with no trajectories during follow-up, neither in terms of temporal variations in exposure nor in terms of changes in residence within the study area of the subjects. However, this should not substantially affect the results of individual exposures (6), although it might limit the possibility to explore the temporal dynamics in the interactions between different environmental stressors and their time-varying health effects (although this was out of the scope of our study). Finally, even the use of exposure variables not referencing the same time period could somewhat influence the results; however, in a long-term study like ours, greater emphasis is placed on spatial contrasts, which remain fairly stable over time (7, 12, 31, 50, 51).

Some important strengths of this study should also be acknowledged. Firstly, we leveraged multiple European cohorts, including large administrative datasets and traditional adult cohorts with rich individual-level information. Secondly, we used a comprehensive codebook for the traditional adult cohorts and implemented harmonized protocols for exposure assessment and statistical analyses for all cohorts. This standardized approach ensured consistency and facilitated the comparison of results. Thirdly, we adopted the exposome framework and employed a dimensionality reduction technique, in this case Principal Component Analysis, and a calculation of an overall risk index, the Cumulative Risk Index. By considering multiple correlated sources of exposure, these methods accounted for the complexity inherent in the exposome concept. They enabled us to capture the cumulative impact of various exposures on mortality and provided a more comprehensive understanding of the interplay between the environment and human health.

In conclusion, this study supports an impact of the external exposome on all-cause mortality. It lays the groundwork for further investigations in the pursuit of more comprehensive evidence regarding the associations and biological mechanisms between combinations of different exposure patterns and human health and, thus, contributes information needed for the future planning of healthier cities.

## Data Availability

The data analyzed in this study is subject to the following licenses/restrictions: The cohort data could not be shared among the EXPANSE project members including named authors, nor can the data be shared externally due to strict national data protection regulations and the General Data Protection Regulation of the EU. Requests to access these datasets should be directed to KH, c.dehoogh@swisstph.ch.
