# Supplementary material for: External exposome and all-cause mortality in European cohorts: the EXPANSE project
Source: Front Epidemiol. 2024 May 28;4:1327218. doi: 10.3389/fepid.2024.1327218 (PMC11165119; doi:10.3389/fepid.2024.1327218)
Supplement: Supplementary file 1 [file Datasheet1.docx]

Supplementary Material

External exposome and all-cause mortality in European cohorts: the EXPANSE Project

Federica Nobile*, Konstantina Dimakopoulou, Christofer Åström, Fabián Coloma, Payam Dadvand, Jeroen de Bont, Kees de Hoogh, Dorina Ibi, Klea Katsouyanni‬, Petter Ljungman, Erik Melén, Mark Nieuwenhuijsen, Regina Pickford, Johan Nilsson Sommar, Cathryn Tonne, Roel C H Vermeulen, Danielle Vienneau, Jelle J Vlaanderen, Kathrin Wolf, Evangelia Samoli, Massimo Stafoggia, *on behalf of the EXPANSE Project Team‬‬‬‬‬‬‬‬‬‬‬‬‬‬‬‬‬‬‬‬‬‬‬‬‬‬‬‬‬‬‬‬‬‬‬‬*

*** Correspondence:** Federica Nobile f.nobile@deplazio.it

**Description of the cohorts**

**Catalonia administrative cohort**

The cohort was built through record-linkage using data collected in the public health administration databases of Catalonia. The public healthcare system covers nearly the entire population (98.8% of the 7.4 million in 2015). Catalonia (32,113 km^2^) is composed of 947 municipalities, grouped in seven health regions (median area 5,425 km2). The cohort includes individual-level (age, sex, comorbidities, smoking status, and income) information and is linked to small area-level socioeconomic data linked to the geocoded residential addresses. Area level SES was measured as PSCA Index, and indicator of deprivation where larger values indicate greater deprivation (https://www.idescat.cat/pub/?id=ist) as well as the proportion of non-Spanish residents at the census tract level in 2018. The original cohort included 5,127,059 adult (≥18 years) residents of Catalonia, who were covered by the public healthcare system in 2015.17 Follow- up in this analysis was until 31 December 2019. The cohort, and its predecessor, have been described in detail elsewhere. We received approval the Parc de Salut Mar Ethics Committee (CEIM-PS MAR, no. 2020/9610).

Participants in dataset

N =3,767,360

Participants without exposure and outcome data

N = 126,766 (3.36%)

Participants with exposure and outcome data

N = 3,640,594 (96.6%)

Participants without covariate data

N = 0 (0.0%)

Participants with complete exposure, outcome and covariate data

N = 3,640,594 (96.6%)

|  | 2015 | 2016 | 2017 | 2018 | 2019 |
| --- | --- | --- | --- | --- | --- |
| Number of all-cause deaths | 31.473 | 32.584 | 34.498 | 40.901 | 43.332 |

*Main references:*

Avellaneda-Gómez C, Vivanco-Hidalgo RM, Olmos S, Lazcano U, Valentin A, Milà C, et al. Air pollution and surrounding greenness in relation to ischemic stroke: A population-based cohort study. Environ Int. 2022;161(November 2021).

Ranzani O, Alari A, Olmos S, Milà C, Rico A, Ballester J, et al. Long-term exposure to air pollution and severe COVID-19 in Catalonia: a population-based cohort study. Nat Commun. 2023;14(1).

**Greece administrative cohort**

The Greek administrative cohort (GRAD, https://www.idika.gr/) is a nationwide population-based cohort. GRAD includes all adults with a social security number aged 37+years in 2014 and follows them until the end of 2019. The cohort contains data on disease diagnoses and related medication subscription and is linked with the mortality registry of the Hellenic Statistical Authority. The administrative cohort includes Individual-level (age, sex, education) information and is linked to small area-level (income, education, unemployment etc.) data from the census 20011 linked to the geocoded residential addresses. The study was approved by the Committee of Ethics and Research Ethics, National and Kapodistrian University of Athens and by the Bioethics Committee of Medical School, National and Kapodistrian University of Athens.

Participants in dataset

N = 6,233,419

Participants without exposure and outcome data

N = 111,998 (1.8%)

Participants with exposure and outcome data

N = 6,121,421 (98.2%)

Participants without covariate data

N = 0 (0.0%)

Participants with complete exposure, outcome and covariate data

N = 6,121,421 (98.2%)

|  | 2014 | 2015 | 2016 | 2017 | 2018 | 2019 |
| --- | --- | --- | --- | --- | --- | --- |
| Number of all-cause deaths | 96,236 | 104,810 | 103,591 | 110,224 | 106,507 | 111,071 |

**Rome administrative cohort**

The Rome Longitudinal Study (RoLS) includes adults aged 30 years and older who were resident in Rome on October 9th, 2011 (the reference day of the Census). The population-based cohort was followed-up until 2019 and the subjects were linked to Health Information Systems available in the Lazio Region through an anonymous identification code. Individual-level (age, sex, marital status, occupation status, education, etc.) and area-level (deprivation index, unemployment rate, etc.) information, through geocoded residential addresses at baseline, is available for each subject. The enrolment and selecting procedures were similar to those described in more detail for the 2001 RoLS.

Participants in dataset

N = 1,546,612

Participants without exposure and outcome data

N = 1514 (0.1%)

Participants with exposure and outcome data

N = 1,545,098 (99.9%)

Participants without covariate data

N = 5314 (0.3%)

Participants with complete exposure, outcome and covariate data

N = 1,539,784 (99.6%)

|  | 2011* | 2012 | 2013 | 2014 | 2015 | 2016 | 2017 | 2018 | 2019 |
| --- | --- | --- | --- | --- | --- | --- | --- | --- | --- |
| Number of all-cause deaths | 4762 | 23,703 | 23,652 | 23,872 | 25,399 | 24,352 | 25,950 | 24,715 | 24,688 |

* October 9^th^, 2011

*Main references:*

Cesaroni G, Badaloni C, Romano V, Donato E, Perucci CA, Forastiere F. Socioeconomic position and health status of people who live near busy roads: the Rome Longitudinal Study (RoLS). Environ Health. 2010 Jul 21;9:41. doi: 10.1186/1476-069X-9-41. PMID: 20663144; PMCID: PMC2918588.

Cesaroni G, Badaloni C, Gariazzo C, Stafoggia M, Sozzi R, Davoli M, Forastiere F. Long-term exposure to urban air pollution and mortality in a cohort of more than a million adults in Rome. Environ Health Perspect. 2013 Mar;121(3):324-31.

Cesaroni G, Venturini G, Paglione L, Angelici L, Sorge C, Marino C, Davoli M, Agabiti N. Differenziali di mortalità a Roma: il ruolo dell’istruzione e dei prezzi immobiliari del quartiere di residenza [Mortality inequalities in Rome: the role of individual education and neighbourhood real estate market]. Epidemiol Prev. 2020 Sep-Dec;44(5-6 Suppl 1):31-37. Italian. doi: 10.19191/EP20.5-6.S1.P031.071. PMID: 33415944.

**Sweden administrative cohort**

The SIMSAM is a register based cohort with all adults in Sweden. For the study all individuals aged above 37 in 2010 was included. The cohort contain data on all individuals up until exclusion (incident, death, migration etc.) or the end of the follow-up period. Individual-level (age, sex, living condition, education, etc.) and area-level (income, education, etc.) information, through geocoded residential addresses for each year, is available for each subject. The Umeå SIMSAM Lab data infrastructure used in this study was developed with support from the Swedish Research Council, the Riksbanken Jubileumsfond and by strategic funds from Umeå University.

Participants in dataset

N = 5,051,463

Participants with exposure and outcome data

N = 4,547,063 (96.8%)

Participants with complete exposure, outcome and covariate data

N = 4,274,326 (85.5%)

Participants without exposure and outcome data

N = 504,400 (3.2%)

Participants without covariate data

N = 272,737 (11.2%)

|  | 2010 | 2011 | 2012 | 2013 | 2014 | 2015 | 2016 | 2017 | 2018 |
| --- | --- | --- | --- | --- | --- | --- | --- | --- | --- |
| Number of all-cause deaths | 33 | 87,867 | 89,821 | 88,079 | 86,610 | 82,240 | 88,125 | 88,891 | 88,805 |

**Switzerland administrative cohort**

The Swiss National Cohort (SNC) is a national longitudinal research platform linking census data with birth, mortality, and emigration data. The SNC was approved by the Ethics Committees of the Cantons of Zurich and Bern. In 2010, Switzerland replaced the classic door-to-door census system with the registry-based census repeated each year. From 2010, deterministic linkage with a unique pseudo-ID (SNC-ID), based on the social security number but cannot be traced back to it, is now used to link the births, mortality and emigration registries to the registry-based census. Swiss TPH received the latest SNC data (for 2010-2019) in July 2021 with all the necessary permissions to conduct analyses. Follow-up from 2011-2019 was used here.

Participants in dataset

N = 4,483,453

Participants with exposure and outcome data

N = 4,427,869 (99.7%)

Participants with complete exposure, outcome and covariate data

N = 4,427,855 (98.6%)

Participants without exposure and outcome data

N = 13,288 (0.3%)

Participants without covariate data

N = 14 (0.0%)

|  | 2011 | 2012 | 2013 | 2014 | 2015 | 2016 | 2017 | 2018 | 2019 |
| --- | --- | --- | --- | --- | --- | --- | --- | --- | --- |
| Number of all-cause deaths | 56,762 | 60,255 | 61,150 | 60,238 | 63,781 | 61,316 | 63,266 | 63,050 | 63,289 |

*Main references:*

Bopp, M., Spoerri, A., Zwahlen, M., Gutzwiller, F., Paccaud, F., Braun-Fahrlander, C., Rougemont, A., Egger, M., 2009. Cohort Profile: The Swiss National Cohort—a longitudinal study of 6.8 million people. Int. J. Epidemiol. 38 (2), 379–384.

Spoerri, A., Zwahlen, M., Egger, M., Bopp, M., 2010. The Swiss National Cohort: a unique database for national and international researchers. Int. J.Public Health. 55 (4), 239–242.

Panczak, R., Galobardes, B., Voorpostel, M., Spoerri, A., Zwahlen, M., Egger, M., 2012. A Swiss neighbourhood index of socioeconomic position: development and association with mortality. J. Epidemiol. Comm. Health. 66 (12), 1129–1136.

**The Netherlands administrative cohort**

The selection of the Dutch national cohort has been described elsewhere (Fischer et al., 2015). In short, in the Netherlands population statistics based on digital municipal registers are combined by Statistics Netherlands (http://www.cbs.nl/en-GB/menu/home/default.htm) into a longitudinal file for each individual registered in the municipal registration. These records start on 1 January 1995. Changes in demographic attributes (e.g., death, residential address, partner status, emigration, region of origin) are updated yearly by adding additional information on the nature and the date of the change. In these files, the individual identification number is replaced by an encrypted unique identification number. This identification number is used to enrich the individual files with information from other central data sources available at Statistics Netherlands. For the EXPANSE study, we shifted the baseline to 1/1/20010 and follow-up to 31/12/2019, selecting all Dutch inhabitants who were 37 years of age or older at baseline. In addition, we included only individuals who lived at the same residential address since baseline and those who moved out of their baseline address but returned back within 92 days.

Participants in dataset

N = 9,315,597

Participants with exposure and outcome data

N = 9,144,497 (98.2%)

Participants with complete exposure, outcome and covariate data

N = 9,144,497 (98.2%)

Participants without exposure and outcome data

N = 171,100 (1.8%)

Participants without covariate data

N = 0 (0%)

|  | 2010 | 2011 | 2012 | 2013 | 2014 | 2015 | 2016 | 2017 | 2018 | 2019 |
| --- | --- | --- | --- | --- | --- | --- | --- | --- | --- | --- |
| Number of all-cause deaths | 133,758 | 133,666 | 138,899 | 139,425 | 137,464 | 145,568 | 147,342 | 148,519 | 151,604 | 150,075 |

*Main references:*

P.H. Fischer, M. Marra, C.B. Ameling, G. Hoek, R. Beelen, K. de Hoogh, et al. Air pollution and mortality in seven million adults: the Dutch Environmental Longitudinal Study (DUELS) Environ. Health Perspect., 123 (2015), pp. 697-704

**CEANS cohort (Cardiovascular Effects of Air Pollution and Noise in Stockholm)**

The CEANS cohort is comprised of four subcohorts: The Screening Across the Lifespan Twin Study (SALT) sampled 7,043 individuals from the Swedish Twin Register born 1958 and earlier, who lived in Stockholm County (Lichtenstein et al. 2006). The Stockholm Diabetes Preventive Program (SDPP) is a population-based prospective study of 7,949 subjects aged 35–54 years (Erikssson et al. 2008). The SIXTY subcohort consists of a random population sample of one-third of all men and women living in Stockholm County turning 60 years between August 1997 and March 1999 (Wändell et al.2007). Lastly, The Swedish National Study of Aging and Care in Kungsholmen (SNAC-K) randomly sampled individuals 60+ years of age from a central area in Stockholm (Lagergren et al. 2004). All participants resided in Stockholm County, Sweden.

Participants in dataset

N = 22,213

Participants with exposure and outcome data

N = 21,404 (96.4%)

Participants without exposure and outcome data

N = 809 (3.6%)

Participants with complete exposure, outcome and covariate data

N = 19,888 (90%)

Participants without covariate data

N = 1,606 (7.2%)

|  | 1997 | 1998 | 1999 | 2000 | 2001 | 2002 | 2003 | 2004 | 2005 |
| --- | --- | --- | --- | --- | --- | --- | --- | --- | --- |
| Number of all-cause deaths | 10 | 9 | 41 | 58 | 58 | 103 | 131 | 173 | 181 |

|  | 2006 | 2007 | 2008 | 2009 | 2010 | 2011 | 2012 | 2013 | 2014 |
| --- | --- | --- | --- | --- | --- | --- | --- | --- | --- |
| Number of all-cause deaths | 180 | 183 | 201 | 216 | 200 | 197 | 185 | 59 | 30 |

*Main references:*

Eriksson AK, Ekbom A, Granath F, et al. Psychological distress and risk of pre-diabetes and Type 2 diabetes in a prospective study of Swedish middle-aged men and women. Diabet Med 2008;25:834–42.

Lagergren M, Fratiglioni L, Hallberg IR, et al. A longitudinal study integrating population, care and social services data. The Swedish National study on Aging and Care (SNAC). Aging Clin Exp Res 2004;16:158–68.

Lichtenstein P, Sullivan PF, Cnattingius S, et al. The Swedish Twin Registry in the third millennium: an update. Twin Res Hum Genet 2006;9:875–82.

Wändell PE, Wajngot A, de Faire U, et al. Increased prevalence of diabetes among immigrants from non-European countries in 60-year-old men and women in Sweden. Diabetes Metab 2007;33:30–6.

**EPIC-NL cohort (European Prospective Investigation into Cancer and Nutrition, the Netherlands)**

The EPIC-NL combines two EPIC-cohorts within the Netherlands: The Monitoring Project on Risk Factors and chronic diseases in the Netherlands (MORGEN) cohort which consists of a general population sample aged 20–59 years from three Dutch towns (Amsterdam, Doetinchem and Maastricht). Prospect is a prospective cohort study among women aged 49–70, residing in the city of Utrecht or its vicinity, who participated in the nation-wide Dutch breast cancer screening programme between 1993 and 1997.

Participants in dataset

N = 38,965

Participants without exposure and outcome data

N = 5,897 (15.1%)

Participants with exposure and outcome data

N = 33,068 (84.9%)

Participants without covariate data

N = 3,640 (9.3%)

Participants with complete exposure, outcome and covariate data

N = 29,428 (76.1%)

|  | 1994 | 1995 | 1996 | 1997 | 1998 | 1999 | 2000 | 2001 | 2002 | 2003 |
| --- | --- | --- | --- | --- | --- | --- | --- | --- | --- | --- |
| Number of all-cause deaths | 3 | 6 | 14 | 32 | 39 | 47 | 41 | 42 | 48 | 76 |

|  | 2004 | 2005 | 2006 | 2007 | 2008 | 2009 | 2010 |
| --- | --- | --- | --- | --- | --- | --- | --- |
| Number of all-cause deaths | 66 | 61 | 88 | 102 | 109 | 109 | 147 |

*Main reference:*

Beulens JWJ, Monninkhof EM, Verschuren WMM et al. Cohort Profile: The EPIC-NL study. International Journal of Epidemiology 2010; 39: 1170–78.

**KORA cohort** **(Cooperative Health Research in the Region of Augsburg)**

KORA is a regional research platform for population-based surveys and subsequent follow-up studies in the fields of epidemiology, health economics, and health care research (https://www.helmholtz-munich.de/en/epi/cohort/kora). This analysis includes two cross-sectional population-representative surveys that were conducted in 1994-1995 (S3 survey) and 1999-2001 (survey S4) in the city of Augsburg and two adjacent rural districts including inhabitants of German nationality aged 25 to 74. Follow-up was conducted in 2011 for S3 and 2013/14 for S4.

Participants in dataset

N = 9117

Participants without exposure and outcome data

N = 355 (3.9%)

Participants with exposure and outcome data

N = 8762 (96.1%)

Participants without covariate data

N = 425 (4.7%)

Participants with complete exposure, outcome and covariate data

N = 8337 (91.4%)

|  | 1994 | 1995 | 1996 | 1997 | 1998 | 1999 | 2000 | 2001 | 2002 | 2003 | 2004 | 2005 |
| --- | --- | --- | --- | --- | --- | --- | --- | --- | --- | --- | --- | --- |
| Number of all-cause deaths | 6 | 22 | 35 | 27 | 36 | 46 | 55 | 74 | 71 | 61 | 64 | 68 |

|  | 2006 | 2007 | 2008 | 2009 | 20010 | 20011 | 2012 | 2013 | 2014 | 2015 | 2016 |
| --- | --- | --- | --- | --- | --- | --- | --- | --- | --- | --- | --- |
| Number of all-cause deaths | 89 | 86 | 100 | 100 | 114 | 118 | 125 | 107 | 135 | 97 | 21 |

*Main reference:*

Holle R, Happich M, Lowel H, Wichmann HE. KORA--a research platform for population based health research. Gesundheitswesen 2005; 67 Suppl 1: S19-S25.

**Table S1.** Data source, temporal resolution and time reference of the exposure factors grouped into the three domains: air pollution, land-built-environment and ambient temperature.

| **Domain** | **Exposure variable** | **Units** | **Data source** | **Temporal resolution** | **Time reference** |
| --- | --- | --- | --- | --- | --- |
| **Air pollution** | PM_2.5_, NO_2_, BC, O_3_ | µg/m^3^ | ELAPSE land-use regression model | Annual | 2010 |
| **Land-built environment** | NDVI | 0 to 1 | Terra Moderate Resolution Imaging Spectroradiometer (MODIS) with 250 m x 250 m | Annual | 2019 |
|  | Impervious surfaces | % | Copernicus Land Monitoring Service within 100mx100m grid cell | Annual | 2015 |
|  | Distance to blue spaces | meters | EU-Hydro map developed by the Copernicus Land Monitoring Service | - | 2011 |
| **Ambient temperature** | Mean temperature | Celsius | European Centre for Medium-Range Weather Forecasts (ECMWF) ERA5-Land | Summer and winter | 2010 |
|  | Standard deviation temperature | SD |  | Summer and winter | 2010 |

Abbreviations: BC, Black carbon; NDVI, Normalized Difference Vegetation Index; NO_2_, nitrogen dioxide; NO_x_, nitrogen oxides; O_3_, ozone during warm month; PM_2.5_, particulate matter with an aerodynamic diameter of less than 2.5 μm; SD, Standard deviation

**Figure S1.** European countries involved in the study (Germany: KORA cohort; Greece: Greek administrative cohort; Italy: Roman administrative cohort; Spain: Catalan administrative cohort; Sweden: Swedish administrative cohort and CEANS cohort; Switzerland: Swiss administrative cohort; the Netherlands: Dutch administrative cohort and EPIC-NL cohort).


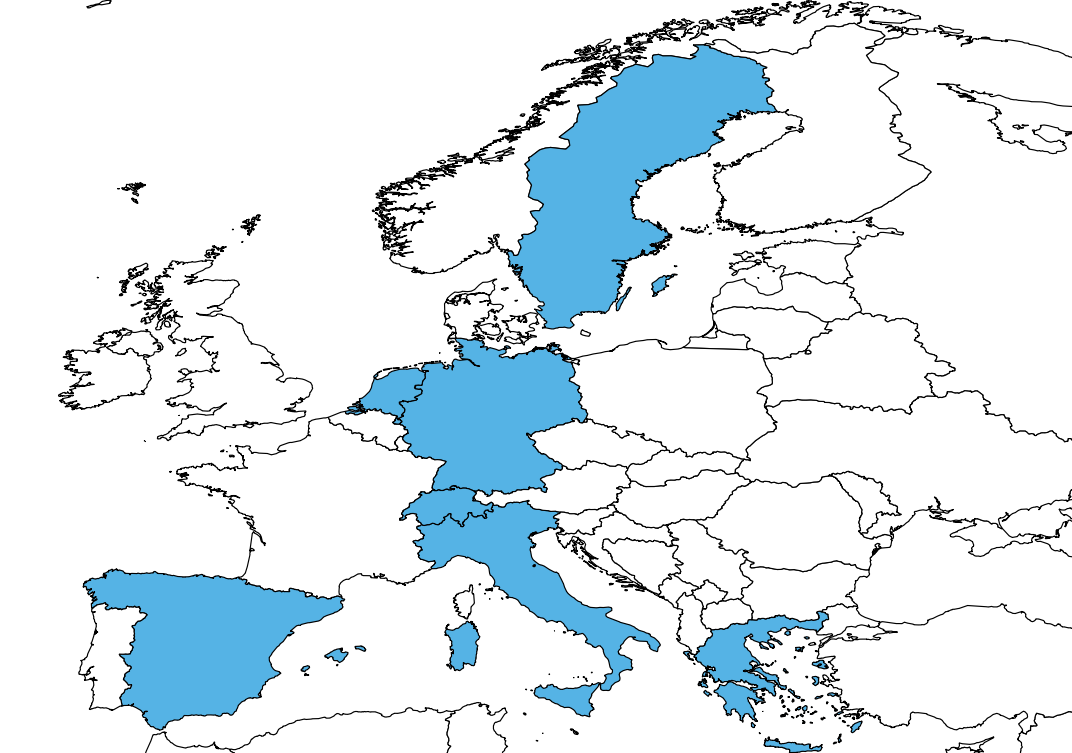


**Figure S2.** Contributions of exposure variables on principal components (dimensions) within each cohort - domain: air pollution.


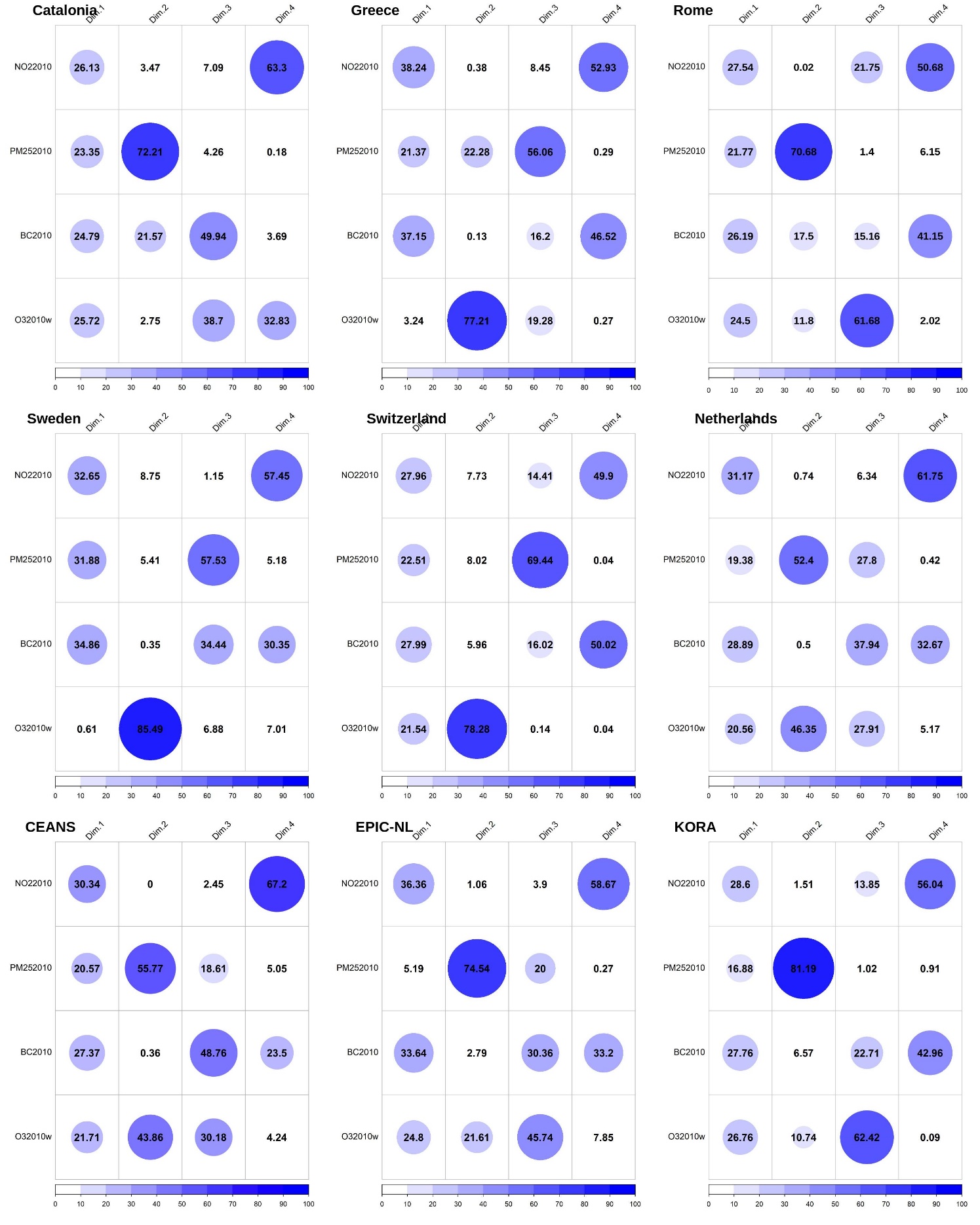


**Figure S3.** Pearson correlation coefficients between exposure variables and principal components (dimensions) within each cohort - domain: air pollution.


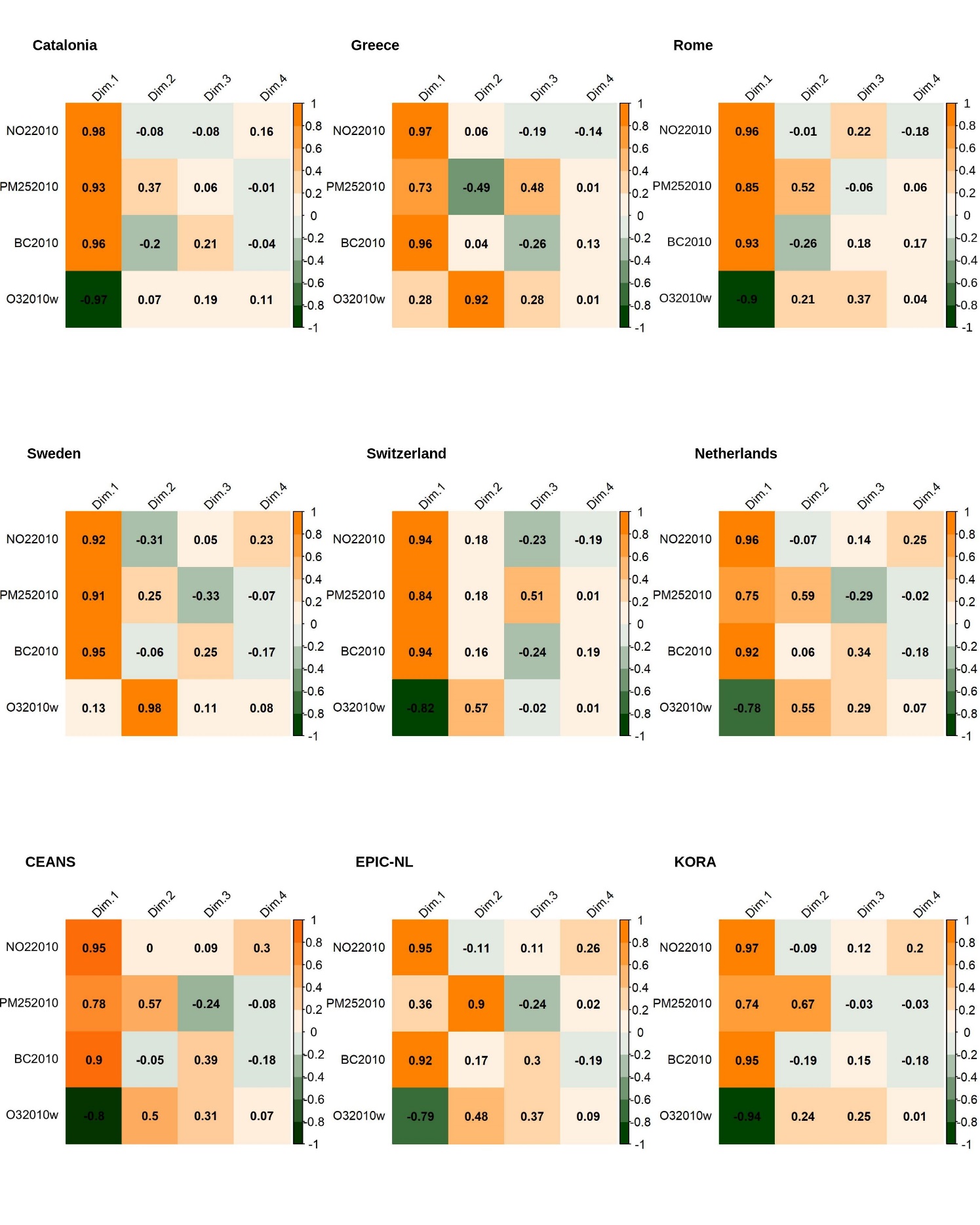


**Figure S4.** Contributions of exposure variables on principal components (dimensions) within each cohort - domain: land-built environment.


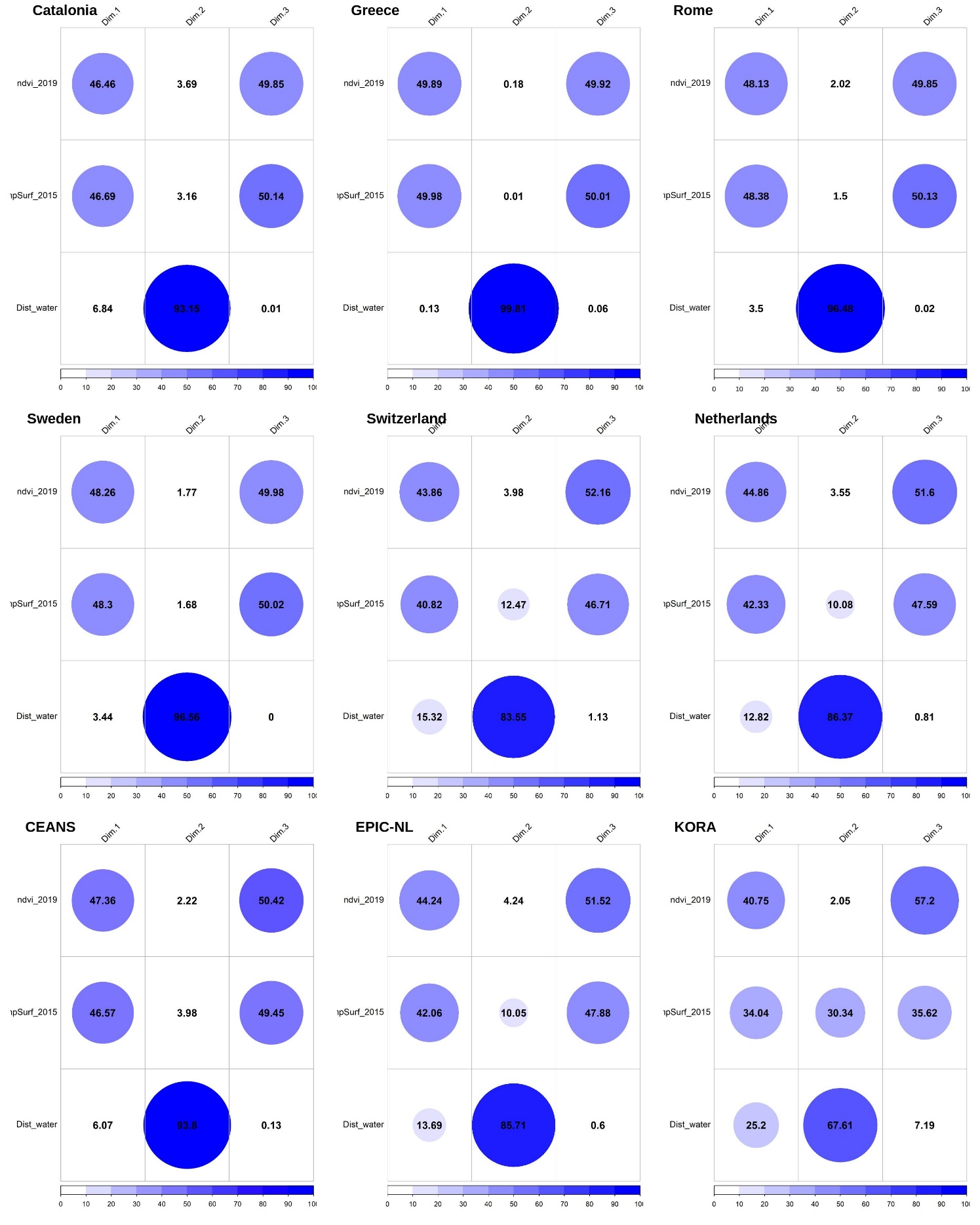


**Figure S5.** Pearson correlation coefficients between exposure variables and principal components (dimensions) within each cohort - domain: land-built environment.


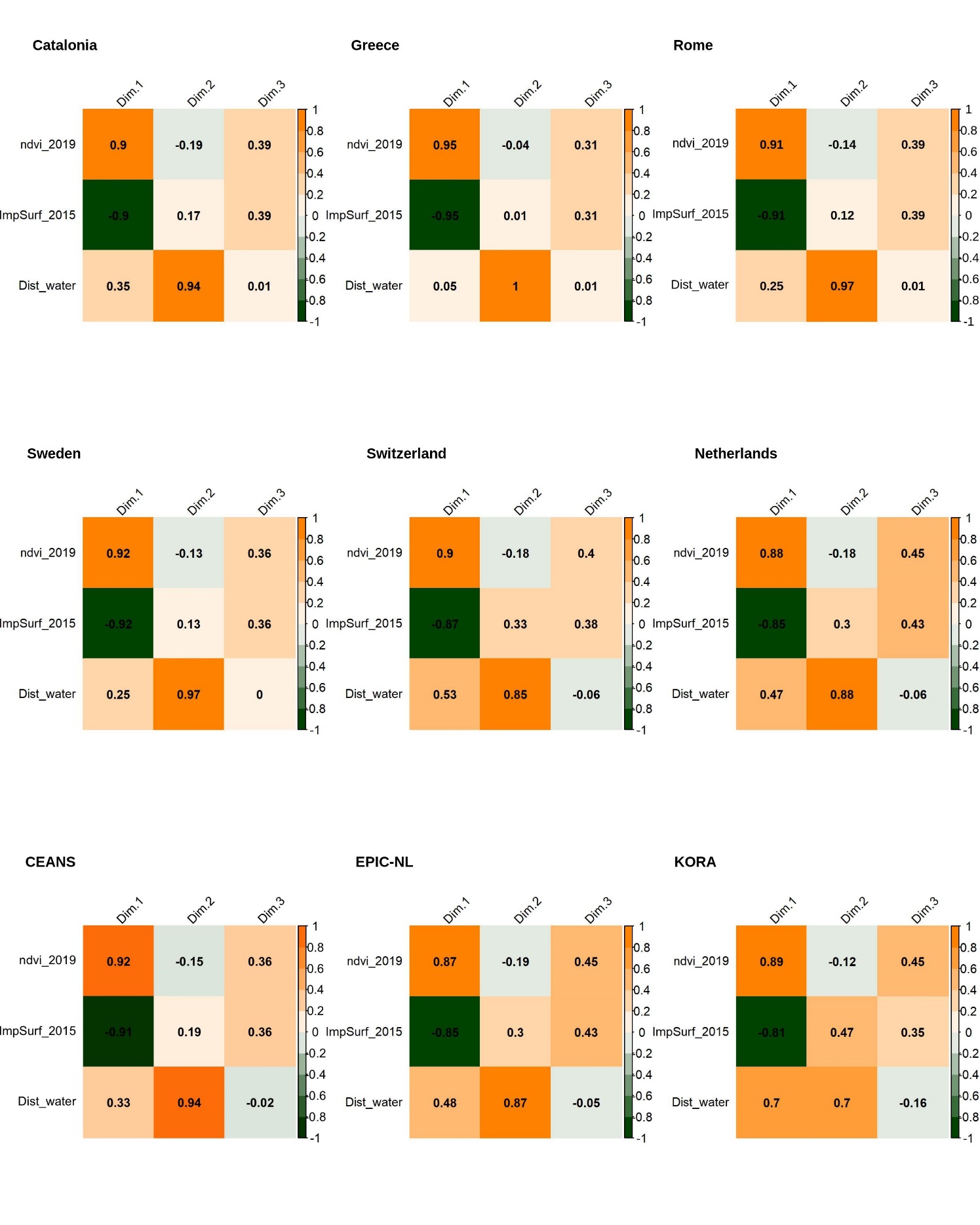


**Figure S6.** Contributions of exposure variables on principal components (dimensions) within each cohort - domain: air temperature.


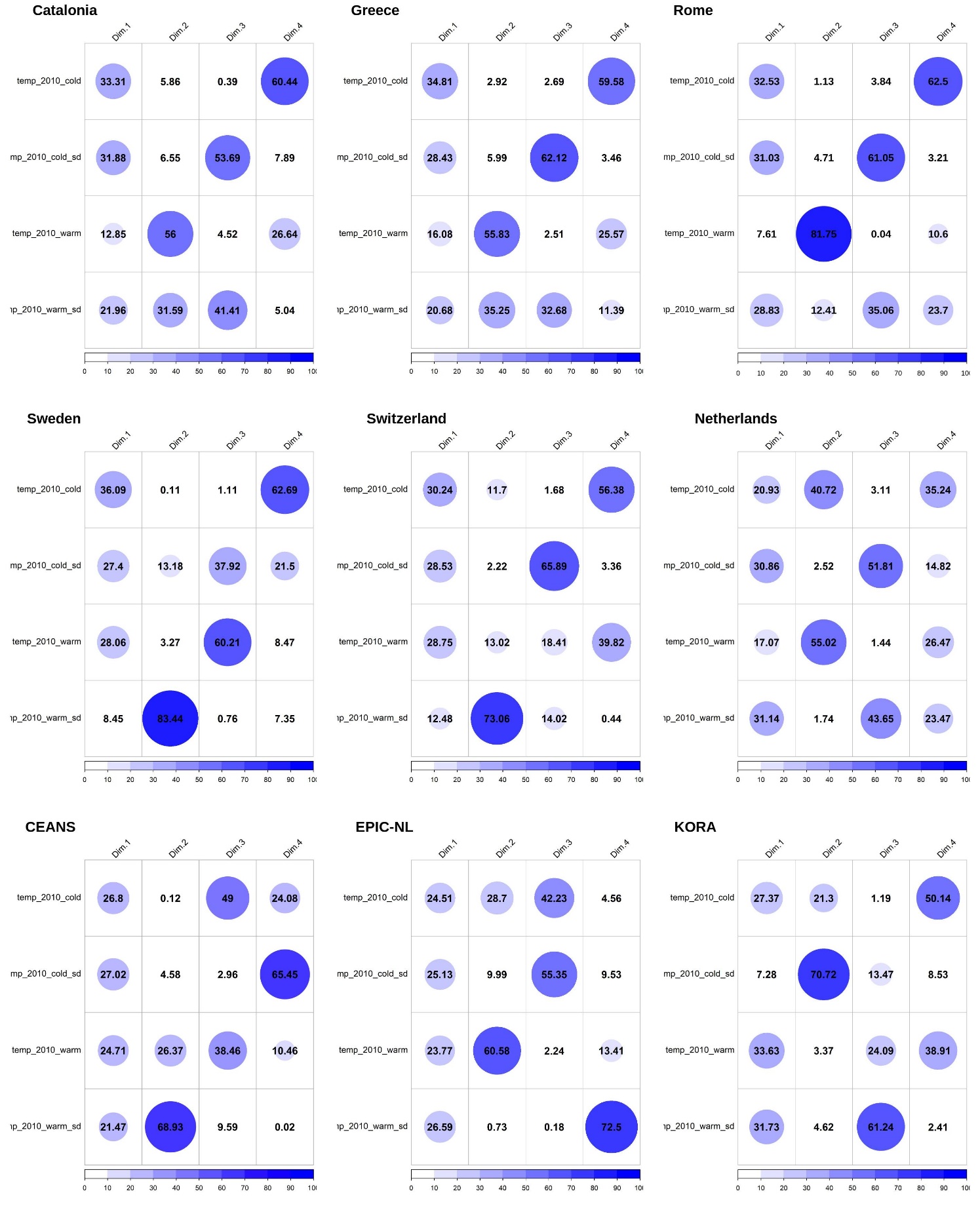


**Figure S7.** Pearson correlation coefficients between exposure variables and principal components (dimensions) within each cohort - domain: air temperature.


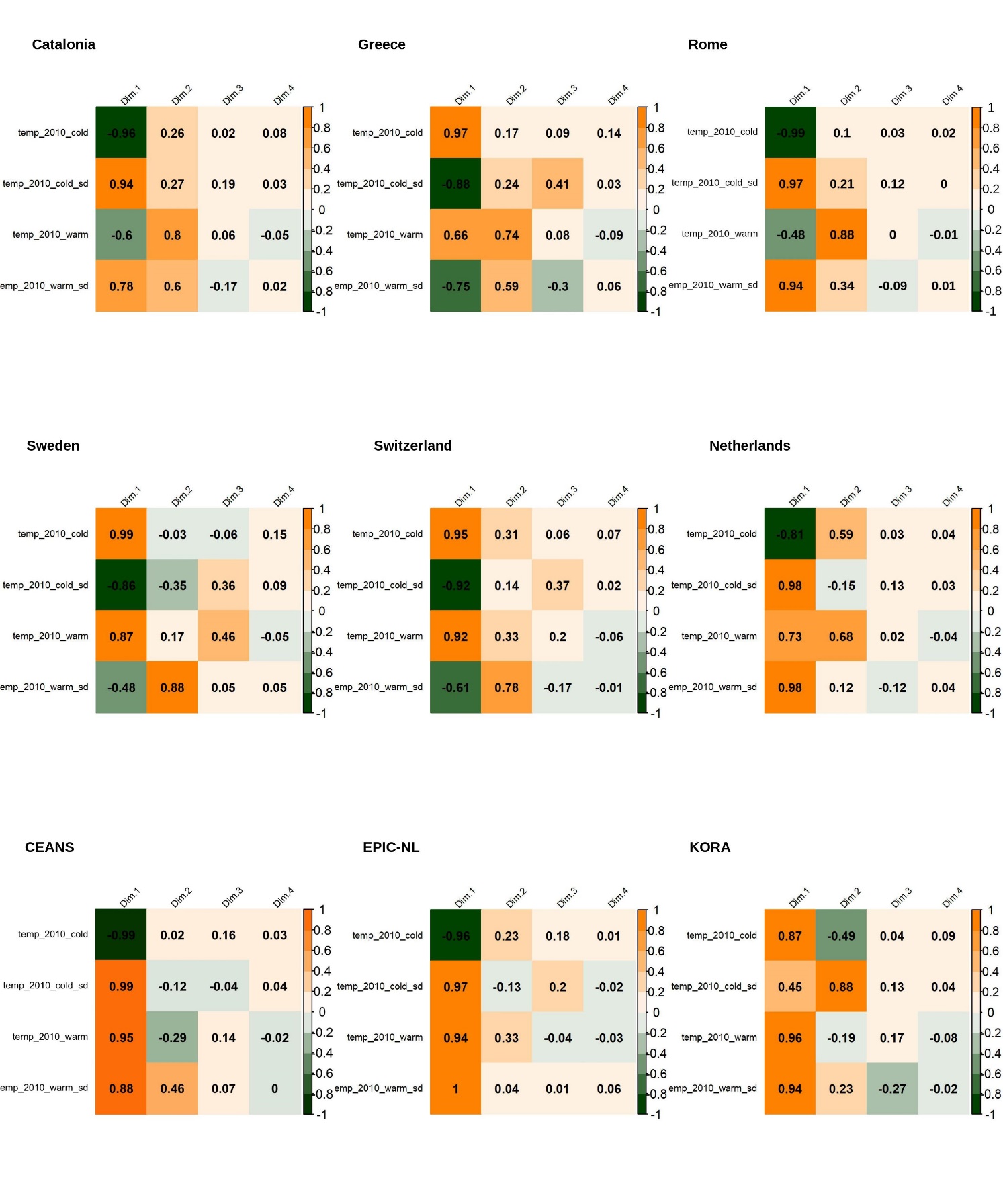


**Figure S8.** Pearson correlation coefficients between selected principal components (PCs) within each cohort.


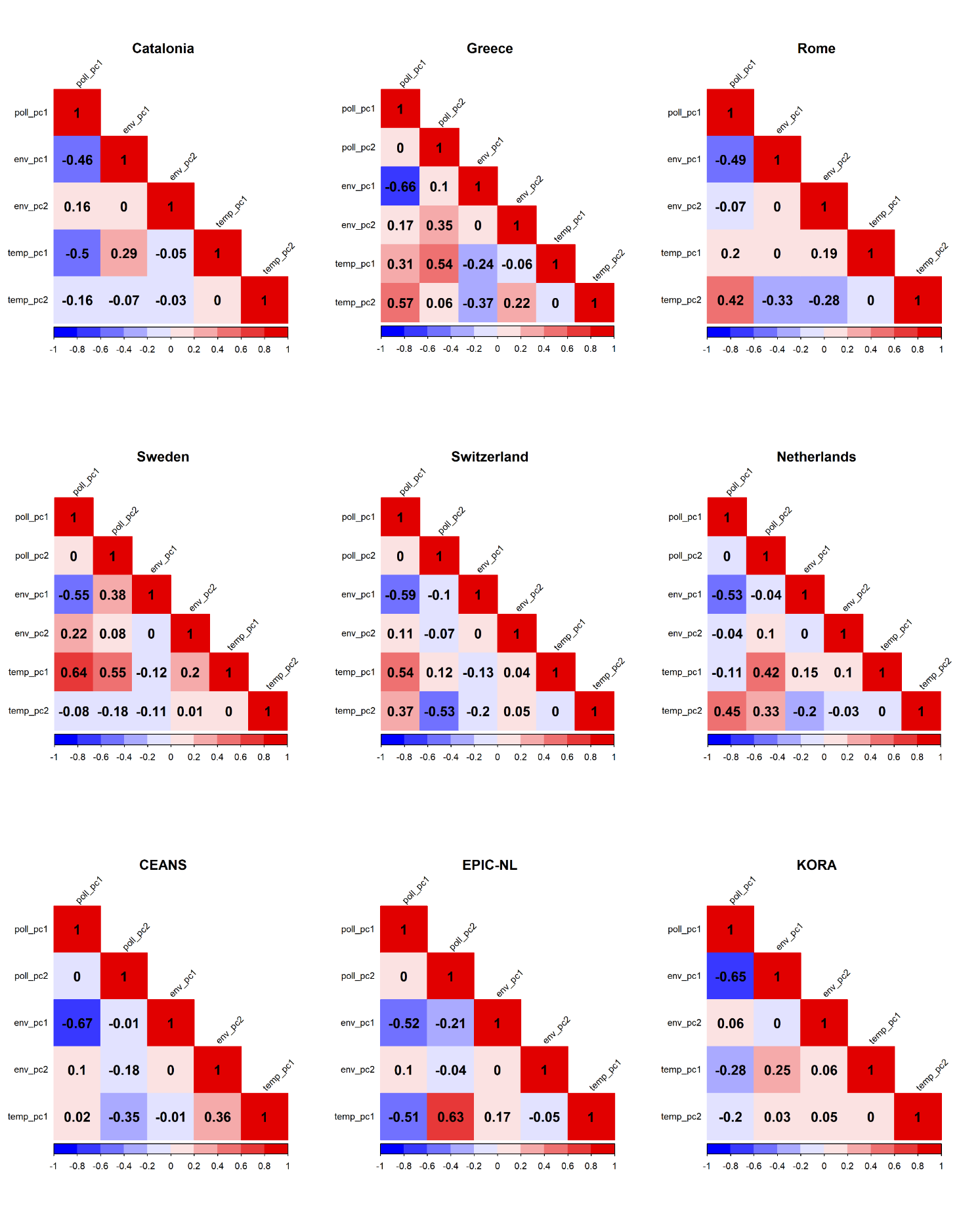


**Figure S9.** Association between principal components (PCs) and all-cause mortality across the European cohorts (six administrative and three traditional adult cohorts) from single-component-exposure Cox proportional hazard models. Hazard Ratios (HRs) with 95% confidence interval (95% CI) per IQR increases. Adjustment details in the footnote.


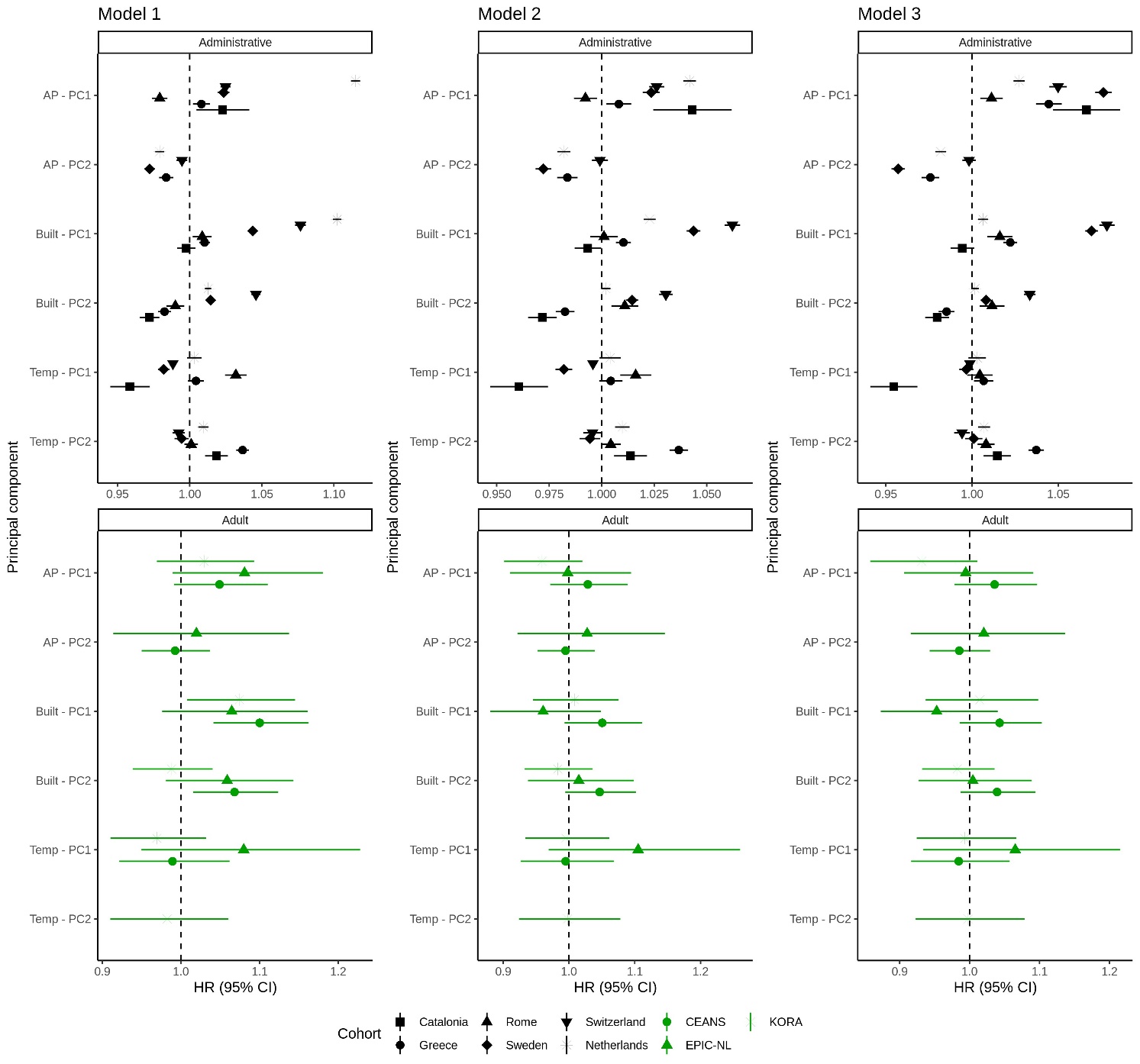


HRs are expressed per IQR increases, except for *Built – PC1* for all cohorts and *Built – PC2* for Rome cohort that are expressed per IQR decreases.

Catalonia

Model 1: age (time scale), sex (strata) and county.

Model 2: additionally adjusted for smoking status, individual income.

Model 3: additionally adjusted for psca index, percentage of non-Spanish residents in census tract, and population density per m^2^.

Greece

Model 1: age (time scale), sex, NUTS1 areas country-wide (4 levels: Attica / Aegean Islands, Crete / North Greece / Central Greece).

Model 2: same as Model 1 since no other individual-level covariates were available.

Model 3: additionally adjusted for tertiary education rate, unemployment rate, degree of urbanicity and married rate. For the Greater Area of Athens and other large municipalities (population greater than 100,000 inhabitants) in Greece, the aforementioned variables were available at square-block level. For the rest of the areas in Greece, the variables were available at municipality unit level.

Rome

Model 1: age (time scale), sex (strata).

Model 2: additionally adjusted for place of birth, education, employment status, marital status, citizenship.

Model 3: additionally adjusted for deprivation index on a census block level and unemployment rate, percentage of graduates and house prices on a neighbourhood level.

Sweden

Model 1: age (time scale), sex (strata).

Model 2: additionally adjusted for living condition, education level.

Model 3: additionally adjusted for district mean income, portion of people with high school or higher education in district, area.

Switzerland:

Model 1: age (time scale), sex (strata), area (i.e. 7 Swiss regions)

Model 2: additionally adjusted for marital status, occupational status,

origin (i.e. Swiss vs. other), language region, socio-economic position index (SEP).

Model 3: additionally adjusted for community-level SEP index and community-level unemployment rate.

The Netherlands:

Model 1: age (time scale), sex (strata), area.

Model 2: additionally adjusted for wealth at 2010, categorized in deciles, partner status at 2010, individual socioeconomical status.

Model 3; additionally adjusted for area-level socio-economic status, area-level mean income at 2010, percentage of low-income households, urbanicity.

CEANS

Model 1: subcohort (strata), age (timescale), sex (strata), and year of baseline visit.

Model 2: additionally adjusted for marital status, body-mass index, smoking (status, duration, intensity, intensity squared), employment status, education.

Model 3: additionally adjusted for area-level socioeconomic status (2001 mean income on a neighbourhood level).

EPIC-NL:

Model 1: subcohort (strata), age (timescale), sex (strata), and year of baseline visit.

Model 2: additionally adjusted for marital status, body-mass index, smoking (status, duration, intensity, intensity squared), employment status, education.

Model 3: additionally adjusted for area-level socioeconomic status (2001 mean income on a neighbourhood level).

KORA

Model 1: subcohort (strata), age (timescale), sex (strata), and year of baseline visit.

Model 2: additionally adjusted for marital status, body-mass index, smoking (status, duration, intensity, intensity squared), employment status, and education.

Model 3: additionally adjusted for area-level socioeconomic status (percentage of households with low income per 5 km² grid cell in 2007).

**Figure S10.** Association between principal components (PCs) and all-cause mortality across the European cohorts (six administrative and three traditional adult cohorts) from two-component-exposure (within domain, where applicable) and multi-exposure (across domains) Cox proportional hazard models. Hazard Ratios (HRs) with 95% confidence interval (95% CI) per IQR increases adjusted for available individual and area-level covariates (model 3). The analyzed domains were air pollution and land-built environment.


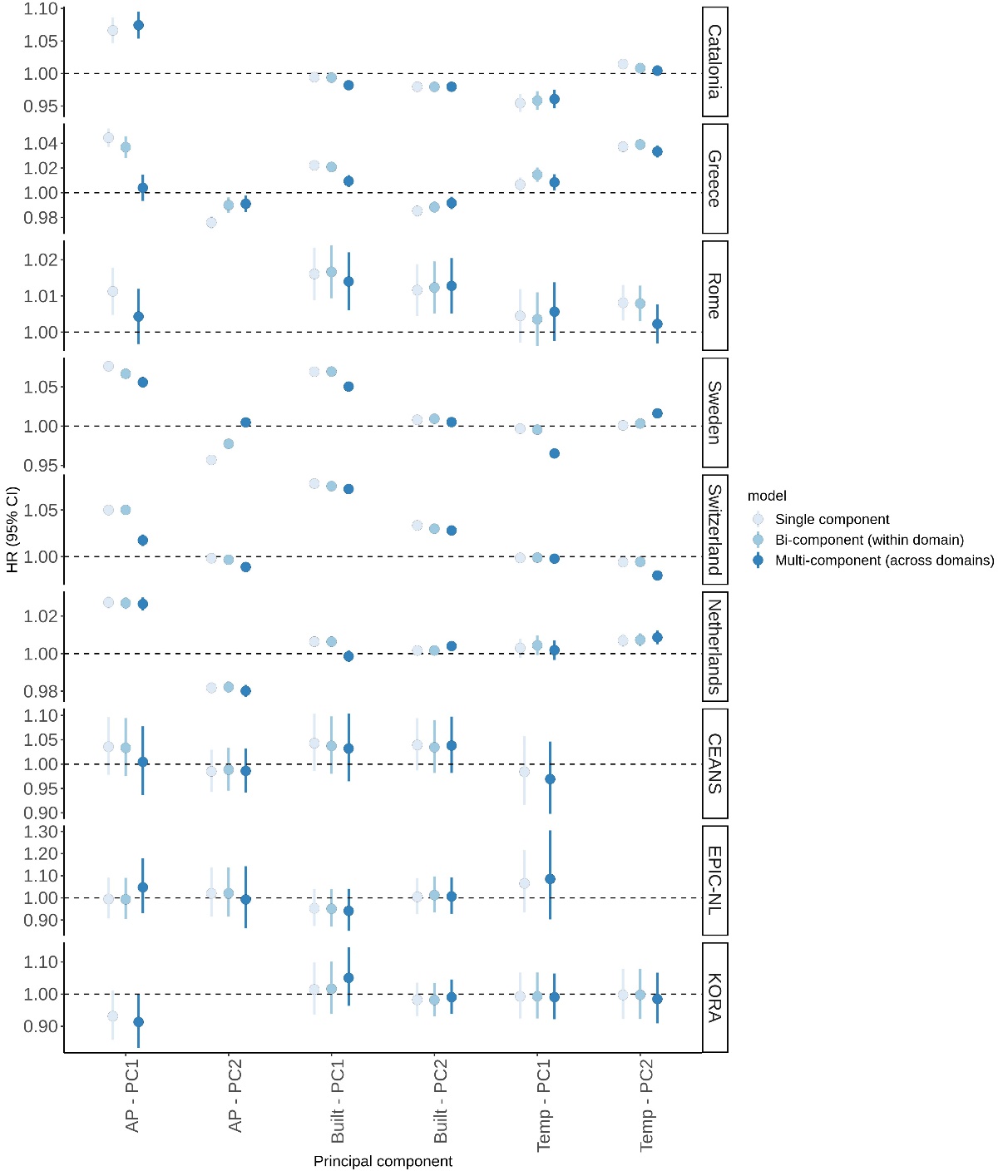


HRs are expressed per IQR increases, except for *Built – PC1* for all cohorts and *Built – PC2* for Rome cohort that are expressed per IQR decreases.

Catalonia: HRs adjusted for age (time scale), sex (strata), smoking status, individual income, psca index, percentage of non-Spanish residents in census tract, and population density per m2.

Greece: HRs adjusted for age (time scale), sex (strata), NUTS1 areas country-wide (4 levels: Attica / Aegean Islands, Crete / North Greece / Central Greece)) & 4 area-level variables: tertiary education rate, unemployment rate, degree of urbanicity in 3 categories: 1. Cities (densely populated areas), 2. Towns and Suburbs (intermediate density areas) and 3. Rural areas (thinly populated areas) and married rate. For the Greater Area of Athens and other large municipalities (population greater than 100,000 inhabitants) in Greece, the aforementioned variables were available at square-block level. For the rest of the areas in Greece, the variables were available at municipality unit level.

Rome: HRs adjusted for age (timescale), sex (strata), place of birth, education level, employment status, marital status, citizenship, deprivation index on a census block level and unemployment rate, percentage of graduates and house prices on a neighborhood level.

Sweden: HRs adjusted for age (time scale), sex (strata), living condition, education level, district mean income, portion of people with high school or higher education in district, area.

Switzerland: HRs adjusted for age (time scale), strata(sex), area (i.e. 7 Swiss regions), marital status, occupational status, origin (i.e. Swiss vs. other), language region, socio-economic position index (SEP), community-level SEP index and community-level unemployment rate.

The Netherlands: HRs adjusted for age (time scale), sex (strata), area, wealth at 2010, categorized in deciles, partner status at 2010, individual socioeconomical status, area-level socio-economic status, area-level mean income at 2010, percentage of low-income households, urbanicity.

CEANS: HRs adjusted for subcohort (strata), age (timescale), sex (strata), and year of baseline visit, marital status, body-mass index, smoking (status, duration, intensity, intensity squared), employment status, education, and area-level socioeconomic status (2001 mean income on a neighborhood level).

EPIC-NL: HRs adjusted for subcohort (strata), age (timescale), sex (strata), and year of baseline visit, marital status, body-mass index, smoking (status, duration, intensity, intensity squared), employment status, education, and area-level socioeconomic status (2001 mean income on a neighborhood level).

KORA: HRs adjusted for subcohort (strata), age (timescale), sex (strata), and year of baseline visit, marital status, body-mass index, smoking (status, duration, intensity, intensity squared), employment status, education, and area-level socioeconomic status (Percentage of households with low income per 5 km² grid cell in 2007).
